# A Rapid Realist Review of Quality Care Process Metrics Implementation in Nursing and Midwifery Practice

**DOI:** 10.3390/ijerph182211932

**Published:** 2021-11-13

**Authors:** Sean Paul Teeling, Carmel Davies, Marlize Barnard, Laserina O’Connor, Alice Coffey, Veronica Lambert, Martin McNamara, Dympna Tuohy, Timothy Frawley, Catherine Redmond, Suja Somanadhan, Mary Casey, Yvonne Corcoran, Owen Doody, Denise O’Brien, Maria Noonan, Rita Smith, Carmel Bradshaw, Sylvia Murphy, Liz Dore, Rosemary Lyons, Máire McGeehan, Anne Gallen

**Affiliations:** 1 Health Sciences Centre, School of Nursing Midwifery and Health Systems, University College Dublin (UCD), 4 Stillorgan Road, Belfield, D04 V1W8 Dublin, Ireland; carmel.davies@ucd.ie (C.D.); 15205785@ucdconnect.ie (M.B.); laserina.oconnor@ucd.ie (L.O.); martin.mcnamara@ucd.ie (M.M.); timothy.frawley@ucd.ie (T.F.); catherine.redmond@ucd.ie (C.R.); suja.somanadhan@ucd.ie (S.S.); mary.casey@ucd.ie (M.C.); denise.obrien@ucd.ie (D.O.); rita.smith@ucd.ie (R.S.); 2 Department of Nursing and Midwifery, North Bank Campus, University of Limerick (UL), Health Science Building, UL, V94 T9PX Limerick, Ireland; Alice.coffee@ul.ie (A.C.); dympna.tuohy@ul.ie (D.T.); owen.doody@ul.ie (O.D.); maria.noonan@ul.ie (M.N.); carmel.bradshaw@ul.ie (C.B.); sylvia.murphy@ul.ie (S.M.); liz.dore@ul.ie (L.D.); rosemary.lyons@ul.ie (R.L.); maire.mcgeehan@ul.ie (M.M.); 3 School of Nursing, Psychotherapy and Community Health, Dublin City University (DCU), 620 Collins Avenue Extension, Whitehall, D09 X984 Dublin 9, Ireland; veronica.lambert@dcu.ie (V.L.); Yvonne.corcoran@dcu.ie (Y.C.); 4 HSE Nursing & Midwifery Planning & Development, Dublin Mid Leinster, Mill Lane, Palmerstown, D20 HY57 Dublin, Ireland; anne.gallen@hse.ie

**Keywords:** quality metrics, nursing, midwifery, implementation, realist, person, outcome measures

## Abstract

Quality measurement initiatives promote quality improvement in healthcare but can be challenging to implement effectively. This paper presents a Rapid Realist Review (RRR) of published literature on Quality Care-Process Metrics (QCP-M) implementation in nursing and midwifery practice. An RRR informed by RAMESES II standards was conducted as an efficient means to synthesize evidence using an expert panel. The review involved research question development, quality appraisal, data extraction, and evidence synthesis. Six program theories summarised below identify the key characteristics that promote positive outcomes in QCP-M implementation. Program Theory 1: Focuses on the evidence base and accessibility of the QCP-M and their ease of use by nurses and midwives working in busy and complex care environments. Program Theory 2: Examines the influence of external factors on QCP-M implementation. Program Theory 3: Relates to existing cultures and systems within clinical sites. Program Theory 4: Relates to nurses’ and midwives’ knowledge and beliefs. Program Theory 5: Builds on the staff theme of Programme Theory four, extending the culture of organizational learning, and highlights the meaningful engagement of nurses and midwives in the implementation process as a key characteristic of success. Program Theory 6: Relates to patient needs. The results provide nursing and midwifery policymakers and professionals with evidence-based program theory that can be translated into action-orientated strategies to help guide successful QCP-M implementation.

## 1. Introduction

Quality measures such as metrics in healthcare promote standardized care, ensuring consistently high quality, safe care. Framework reports published by the Department of Health, and the Health Service Executive in Ireland [1,2,3], which draw on international literature on evidence-based Quality Care Process Metrics (QCP-M), indicate that when effectively implemented, they provide a framework to identify gaps in care delivery. They also enable action planning for quality improvement and offer a mechanism by which care providers can be accountable for the quality of their care delivery [1,2,3]. This paper focuses on understanding the dynamics of implementing specific nursing and midwifery Quality Care Process Metrics (QCP-M), a complex intervention that operates across multiple clinical organizations and care sectors across the Health Service Executive (HSE), to measure and evaluate nursing and midwifery care process outcomes.

The implementation of QCP-M is compounded by the complexity of healthcare organizations, one of the most complex forms of human organization to manage [4], and the multifaceted and dynamic nature of health service delivery [5]. Given this complexity, interventions such as QCP-M rarely work in the same way in different contexts [5]. The reality is that effective healthcare interventions can have their impact mitigated by the conditions of the healthcare context [5,6,7]. Implementation and context are inextricably bound.

Contextual influences explain much of the variation in implementation efforts and their levels of success. They describe a set of circumstances or unique factors that surround a particular implementation effort and take account of the broader systemic context and the specific setting in which an intervention is implemented [8,9]. Context generates multiple sources of contingency and a wide variety of confounding factors that directly impact implementation [10]. These include historical and cultural influences, political and social contexts, economics and resources, organizational structure, leadership, and professional and patient behavior [8,11,12]. Context is also dynamic and bound by its setting; therefore, what might constitute a contextual barrier in one environment may be an enabler in another [8,13]. This explains why some interventions flourish in some practice settings and languish in others [9].

The complex, context-dependent nature of implementing interventions has directed attention to examining context. Evaluations of an intervention must consider the implementation process and acknowledge the broader social structural context within which clinicians, patients, and practices operate [10]. Conventional systematic reviews to determine the evidence of whether interventions work (or not) often result in limited answers [14]. It is not sufficient to know if an intervention is effective; it is essential to understand why the intervention works, how, for whom, and in which contexts. Therefore, understanding context across multiple settings helps us to better understand how implementation processes might lead to ‘scaling up’ and scaling out between settings which are critically important for a national implementation initiative, such as the implementation of specific Nursing and Midwifery QCP-M within healthcare systems. Evaluating the implementation of complex interventions in healthcare practice is therefore important for policymakers and implementation planners as it provides a theoretical framework to guide the process [10].

This study is informed by the RAMESES II (Realist And Meta-narrative Evidence Syntheses: Evolving Standards) reporting guidelines for realist evaluations [15]. A study protocol for conducting this Rapid Realist Review (RRR) was published in January 2021 (version 2) and outlined the study aim, which is to conduct an RRR through a synthesis of the international literature (published and grey) that generates program theories to improve understanding of facilitating and constraining influences related to the implementation of nursing and midwifery QCP-M [16]. A RRR fundamentally improves understanding of how programs work within specific contexts and what conditions may impede or develop successful outcomes through iteratively structuring the empirical and theoretical literature [17]. This flexible methodology seeks to explain generative causation within social settings by identifying specific patterns of interaction and addressing the basic question of ‘what is it about this intervention that works in this context and why?’ [14]. RRR was developed by Pawson [14] to examine existing data to better understand complex problems. Wong and colleagues [15] suggest that complex problems comprise:

- Numerous interacting components within an intervention.
- Numerous behaviors demonstrated by those involved in an intervention.
- Number of groups involved in an intervention.
- Variability and number of outcomes.
- Allowance for flexibility.
- Non-linear patterns.
- Reliance on people.
- Context dependency.

Pawson and colleagues [14] claim that the differences between a standard meta-analysis approach and a realist approach are significant. Meta-analysis looks at program effects, measuring effect sizes and seeking mean effect through a systematic review. However, realist understanding sees programs as theories, tests theories, and uses systematic review to synthesis theory [14]. This understanding was fundamental to our choice of RRR for this study.

## 2. Materials and Methods

### 2.1. Evaluation Design

In 2020, due to COVID-19 restrictions, the research team adopted a virtual technological approach to conduct the RRR. To undertake the review, a local reference panel and an expert panel (Supplementary File S1) were established. The local reference panel consisted of eight key intervention stakeholders and healthcare professionals as knowledge users, who will utilize the results of this RRR to develop further and implement the intervention of the QCP-M in clinical practice. An expert panel consisting of health systems researchers from three Irish universities, with combined expertise in nursing, midwifery, implementation science, quality improvement, and critical realism, assisted in defining the research questions, reviewed the inclusion and exclusion criteria and tailored the search strategy for clarity and consistency, contributed to the synthesis of findings and verified interpretation of results. The inclusion of key stakeholders and knowledge users as part of the review process increased the relevance, clarity, and awareness of the review findings’ transferability [18,19].

**Figure 1:**
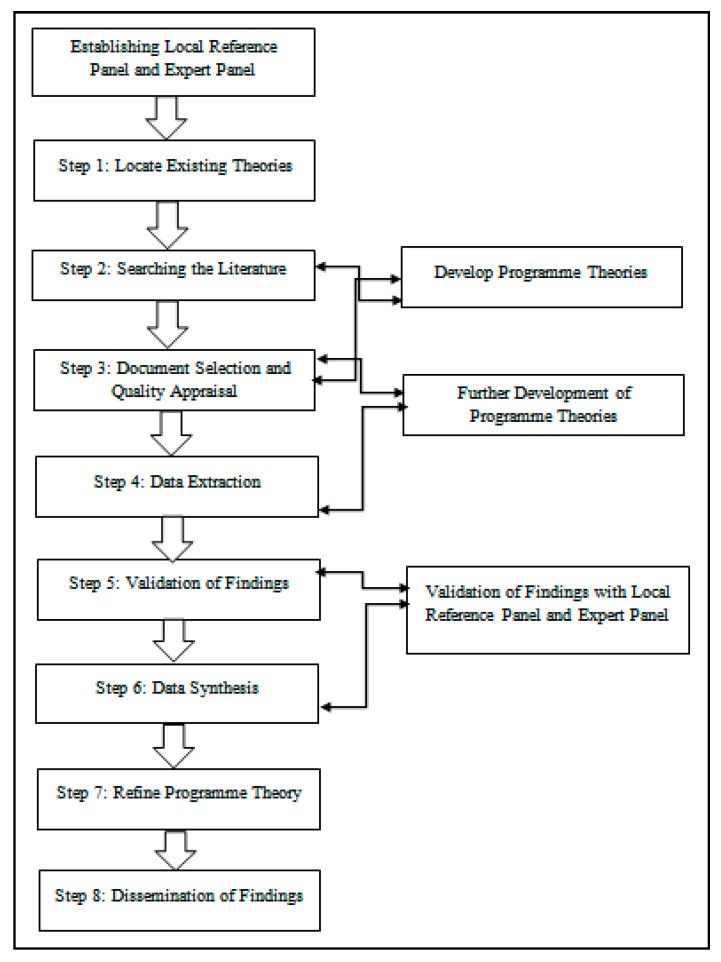
Review design.

The RRR was conducted over six months from February to July 2021. The multifaceted review design involved an eight-step approach [20], based on a collation of the five review stages promoted by Pawson [21], a previously drafted protocol depicting access to high-quality primary care for older people [22], and a project diagram design [23]. The iterative eight-step design approach includes the location of excising theories, searching the literature, document selection, quality appraisal, data extraction, validation of findings, data synthesis, refinement of the initial program theory, and the dissemination of the review findings. The eight-step approach is illustrated in Figure 1.

### 2.2. Locate Existing Theories by Searching the Literature

Pertinent to the literature review was identifying existing theories that successfully implemented a suite of QCP-M relevant to nursing and midwifery practice. For the researchers seeking to understand context, an RRR emphasizes the importance of understanding and explaining context-specific circumstances and the mechanisms that lead to the outcome of an intervention, the intervention, in this case, being the QCP-M. The research team agreed on the scope of the RRR to answer the question, ‘What factors enable the successful implementation of a suite of quality care process nursing and midwifery metrics (the intervention) across all areas in nursing and midwifery practice?’. Additional sub-research questions were, ‘In nursing and midwifery quality care process metrics, what contexts and mechanisms lead to positive implementation outcome?’, also, ‘In nursing and midwifery quality care process metrics, what contexts and mechanisms lead to negative implementation outcomes?’ and ‘What were the dominant outcome patterns in identified contexts?’

With the assistance of a college librarian, a search strategy was designed and conducted involving a two-step approach. Firstly, a preliminary background search was conducted in PubMed Central and Excerpta Medica Database (EMBASE) to identify the keywords, subject headings, and alternative terminology associated with the topic to guide the forming of the second search strategy. Specific Boolean operators such as AND, OR, NOT were used to define the search, and truncation markers were applied. An inclusion and exclusion criteria were developed and applied (see Table 1) All searches were limited to the English language, involving human participants of all age groups with an available abstract dating from 1 January 2010 to 31 July 2020. Articles sourced were reviewed and interrogated for the development of an initial program theory related to the successful implementation of interventions such as QCP-M for further refinement during the RRR process. Secondly, seven electronic databases were searched, including EMBASE, PubMed Central, The Cumulative Index to Nursing and Allied Health Literature Complete (CINAHL Complete), APA PsycINFO, Applied Social Sciences Index, and Abstracts (ASSIA), and Cochrane Database of Systematic Reviews (CDSR). Grey literature published from 1 January 2010, to 31 July 2020 in the following databases was also included, Lenus Irish Health Repository, Open Grey, Virginia Global e-Repository, and Clinical Trials.Gov.

A PICO framework was adopted for the structuring of the keywords (see Table 2). In the PICO framework: (P)—Population refers to the sample of subjects. Here, the ‘P’ refers to nurses and midwives. (I)—Intervention refers to the treatment or intervention that will be provided, which is the Quality Care Process Metrics. (C)—Comparison identifies a reference group for comparison, control group, or the study design. No comparison group was identified. (O)—Outcome represents what results are to be measured to examine the effectiveness of the intervention. Furthermore, it relates to the research question: What factors enable the successful implementation of a suite of quality care process nursing and midwifery metrics across all areas of nursing and midwifery practice?

The initial high-level program theory identified, suggests that the intervention of Quality Care Process Metrics (QCP-M) in clinical practice has a positive impact on nursing and midwifery care processes. This initial theory described how the intervention of QCP-M is expected to work.

### 2.3. Document Selection

Results from database searches were inputted into Covidence [24]. The initial title and abstract screening through Covidence were completed by four members of the research team, divided into two groups (AC and MC) and (SS and CR). The local reference panel searched the grey literature for inclusion or exclusion, and any title or abstract conflicts were reviewed in Covidence by a RE methodology expert (SPT) and resolved. Four teams comprising of two to three reference panel members each conducted a full-text screening in Covidence (OD, RL, and CB), (LO’C and MN), (DOB and RS), and (SS and CR). Likewise, full-text screening conflicts were reviewed by an expert in RE methodology (SPT) and resolved.

### 2.4. Quality Appraisal

The Crowe Critical Appraisal Tool (CCAT) was utilized for quality appraisal of the initial 68 studies included. Three researchers (LO’C, YC, and MB) appraised the selected 68 studies for their level of quality. A standardized approach was adopted, and each study was mapped using the CCAT Form (v1.4) [25] following the allocated study identification number provided in Covidence. Both the individual category scores and the total score, as recommended by the CCAT, were included within the final decision for study inclusion. Requested guidance from the CCAT author, specifically concerning the mapping of systematic reviews, provided valuable feedback and was applied. All systematic reviews were mapped according to the apparent themes described and the strength of the evidence supporting each theme. Themes were arranged from those displaying the most evidence to those displaying the least evidence. Application of the CCAT reduced the initial 68 studies selected for inclusion to 37, which were included for data extraction, synthesis, and analysis.

### 2.5. Data Extraction, Synthesis, and Analysis

A framework was designed to guide the data extraction activities, and a data extraction tool was generated to tabulate the findings from the included papers. Expert panel meetings occurred regularly, and panel members were divided into three teams to extract CMO-configurations (CMOc) from the selected papers (SPT, MM, AC, and MB), (CD, DT, and TF), and (VL, SS, and CR). During panel meetings, CMOc of individual included papers were discussed by all members and exhausted through consensus. The final review of themes was entered into NVivo 12, a specialized qualitative software, to facilitate coding and thematic analysis of the data. Panel members participated in a final adjudication of approved CMOc and themes. Following the final adjudication, the final agreed coding was completed in NVivo 12.

To facilitate understanding of the evidence generated during the RRR, the CMOc identified were mapped to existing implementation theory using the Consolidated Framework for Implementation Research (CFIR). Damschroder and colleagues (2009), developed the CFIR, which identifies constructs across five multilevel domains that influence effective intervention implementation and effectiveness. This framework assisted the interpretation of the review findings as it facilitated a systematic evaluation of multilevel implementation contexts and helped identify factors that may have influenced intervention implementation and effectiveness. The CFIR is a well-validated framework for implementation evaluation, offering an organizational framework for synthesizing and building knowledge about what works, where, across multiple settings [8,26].

The linking of the CMOc to the CFIR (Supplementary File S2) enabled us to connect the program theories to an evidence-based framework that focuses on intervention implementation within the healthcare context, which, as already outlined, is multifaceted and complex [4,27]. The analysis enables the translation of generic constructs from implementation theory into context-dependent narratives that can inform policymakers in the direction required to support implementation.

## 3. Results

### 3.1. Data Analysis

**Figure 2:**
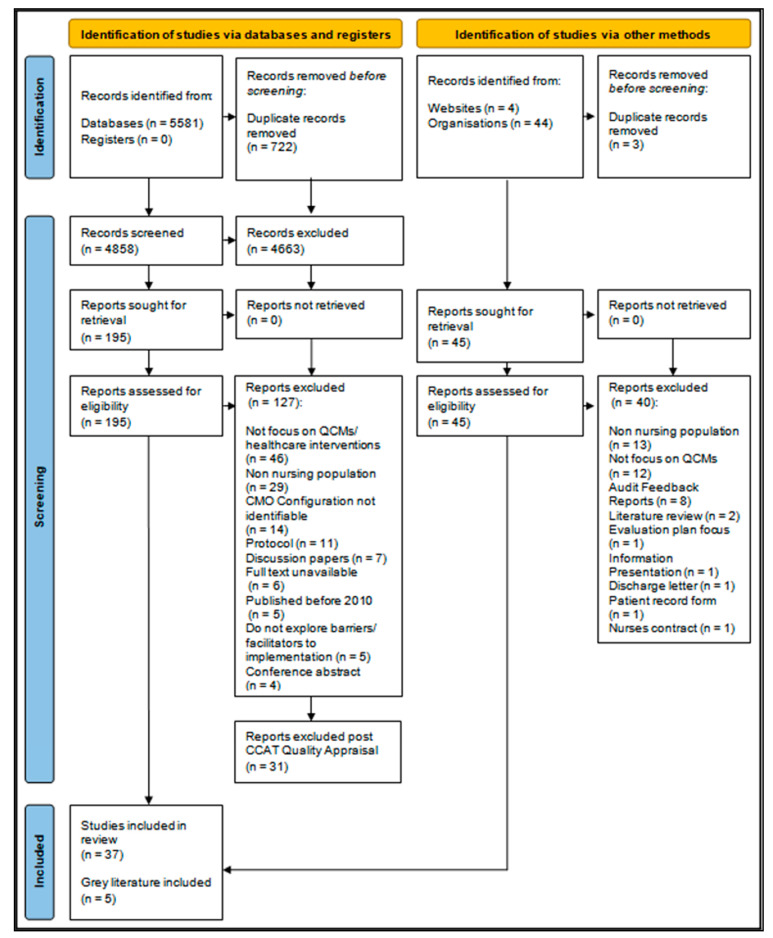
Modified PRISMA diagram.

We identified 5581 references across all search methods; of these, we identified 722 duplicates for removal. Titles and abstracts of 4858 documents were then reviewed, leading to a full-text review of 195 documents. Of these, 127 documents were removed, and 31 additional documents were removed post the CCAT quality appraisal process. A total of 37 documents were included in the RRR and enabled the refinement of the initial CMO hypotheses. This constituted 37 documents from our database search, fully outlined in the Supplementary File (S3), and five documents from a grey literature search [1,3,28,29,30] (Figure 2).

Document characteristics included 21 primary research studies, 3 quality improvement case studies, and 13 others, such as reviews or syntheses. Of the 37 documents, 25 referred to nursing, two to midwifery, and six to both nursing and midwifery. Of these, 28 referred to policy, with twelve referring to specific quality measurement initiatives.

### 3.2. Initial Programme Theories

**Figure 3:**
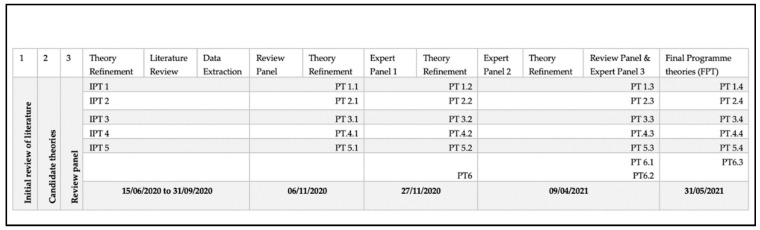
Iterative development of program theory.

The literature review related to the research question ‘what factors enable the successful implementation of a suite of nursing/midwifery Quality Care Process Metrics (QCP-M) across all areas in nursing and midwifery practice’ facilitated Initial Programme Theory (IPT) development relating to the QCP-M. Specifically, congruent with realist methodology, it enabled an understanding of what about the QCP-M works, for whom, in what circumstances, and why [31]. As indicated in Figure 3, five IPTs were developed that we mapped to existing implementation theory in the CFIR [8], which has been associated with effective implementation. Each IPT is elaborated below with specific Context (C), Mechanism (M), and Outcomes (O) explicated.

#### 3.2.1. IPT1 Specific Characteristics of the QCP-M

In introducing the intervention of QCP-M into clinical sites, the source (internal or external), design of and evidence base for the intervention (C) influence how staff interact with its deployment (M), leading to variation in anticipated outcomes from the QCP-M (O).

#### 3.2.2. IPT2 External Factors Influencing QCP-M Implementation

The degree to which the clinical site’s quality metric goals are aligned with other external organizations that influence policy, guidelines, and benchmarking (C) influences how the organization implements the QCP-M (M), resulting in differing experiences of and outcomes from the intervention (O).

#### 3.2.3. IPT3 Internal Factors Influencing QCP-M Implementation

The existing cultures and systems within clinical sites are facilitative of quality interventions (C) and support practice cultures and practice settings that nurses and midwives engage with (M), impacting the state of readiness of the clinical site for sustainable implementation of the QCP-M (O).

#### 3.2.4. IPT4 Individuals’ Perceptions of QCP-M Implementation

Nurses and midwives have their own perceptions and beliefs about QCP-M (C), influencing how they react to their introduction (M), leading to variation in QCP-M adoption and outcomes (O).

#### 3.2.5. IPT5 The Process of Engaging Nurses and Midwives in QCP-M Implementation

The successful introduction of QCP-Ms into a clinical site is dependent on the quality of individual sites’ staff engagement (C) and staff being adequately supported by change champions or implementation leads (M) that influence the degree of success of the QCP-M implementation (O).

### 3.3. Theory Refinement

The developed IPTs were subjected to iterative adjudication by both the review panel and the expert panel who facilitated IPT development through confirming, refuting, or refining developed CMOc [15]. Figure 3 demonstrates the iterative adjudication of the five ITP leading to a final set of six program theories. Literature relevant to the iterative development of each program theory is cited as relevant, and CMOc and their data sources are detailed in Supplementary File S4.

### 3.4. Finalised Theories

#### 3.4.1. Programme Theory 1: Evidence Base and Accessibility of the QCP-M

Program theory 1 focuses on the evidence base of QCP-M and their ease of use by nurses and midwives working in busy and complex care environments. The program theory is supported by five CMOc extracted from seven literature sources.

*The source and evidence base of QCP-M is important to nurses and midwives working in diverse clinical settings. These factors, in addition to the degree of complexity of QCP-M and their adaptability to a local context, influence how nurses and midwives interact with, trial, and use them and can lead to successful implementation and use of the QCP-M [32,33,34,35,36,37]*.

The scientific evidence base of QCP-M [33,34,36,37] and their source [36,37] is considered important by nurses and midwives and influences their attitudes to their use. QCP-M that are uncomplicated and adaptable to trial and use in local contexts facilitate successful program implementation and outcomes [32,34,35,36].

#### 3.4.2. Programme Theory 2: The Influence of External Factors on QCP-M Implementation

Program theory 2 is supported by two CMOc extracted from five literature sources. The program theory addresses external strategies to spread interventions such as national policy and regulations and the degree to which clinical sites are outward-facing.

*Clinical sites that are outward-facing and well-networked are adept at introducing and implementing external strategies (including policy, regulation, guidelines, and QCP-M) and using available resources to spread the intervention. Nurses and midwives in these sites engage with provided resources (such as champions and materials), resulting in an increased chance of intervention success [33,36,38,39,40]*.

External collaboration with networks regionally and locally [36] reduces the possibility of variance in the implementation of strategies such as QCP-M [33], increasing the likelihood of an evidence-based approach [33] in a standardized way [38]. Where interventions are supported at a national level [38] by an implementation framework [40] and organizations are externally facing, then there is a more standardized approach to implementation [33], leading to successful adoption of the intervention.

#### 3.4.3. Programme Theory 3: Existing Cultures and Systems within Clinical Sites

Program theory 3 comprises 5 CMOc extracted from 24 literature sources. The program theory addresses how existing cultures and learning climates within clinical sites reflect their readiness for QCP-M implementation.

*Clinical sites that show readiness for QCP-M implementation have strong internal networks and communication systems that facilitate a practice culture compatible with the introduction of quality interventions and a learning climate supportive of nurses and midwives who are implementing the QCP-M, resulting in an increased chance of the intervention becoming embedded in practice [34,36,38,39,40,41,42,43,44,45,46,47,48,49,50,51,52,53,54,55,56,57].*

Clinical sites that have effective internal networks and communication systems [34,41,44,46,50] and an organizational commitment to, and readiness for change implementation [40,42,43,44,45,48,52,53,55,57] evidence practice cultures that are supportive of staff [32,38,40,41,42,43,47,50,53] and compatible with quality interventions [38,39,40,41,44,47,48,57] in a supportive learning climate [36,41,42,43,45,46,48,49,54,55,56,57] leading to the successful adoption and integration of the QCP-M into clinical practice.

#### 3.4.4. Programme Theory 4: Nurses’ and Midwives’ Knowledge and Beliefs

Program theory 4 focuses on the particular knowledge set, values, and beliefs of individual nurses and midwives. It is supported by two CMOc extracted from eleven literature sources.

*The implementation of QCP-M in clinical sites is influenced by nurses and midwives, whose local knowledge, values, beliefs, and individual characteristics determine their interaction with the intervention. Awareness of this facilitates the successful implementation of the QCP-M within the clinical setting [34,39,41,44,45,51,54,58,59,60,61]*.

Clinical sites recognise and respect the knowledge [34,39,41,45,54,58,61], beliefs [36,44,54,61], and values [45] of nurses and midwives and their unique individual characteristics [41,51,58,59,60], thereby facilitating the promotion and adoption of quality improvement initiatives [61].

#### 3.4.5. Programme Theory 5: Engaging Nurses and Midwives in QCP-M Implementation

Three CMOc extracted from sixteen included papers support program theory 5. The program theory relates to the factors that influence nurse and midwife engagement in the implementation of QCP-M.

*QCP-M introduced into clinical sites that have supporting education and training functions, and champions/implementation leads to support them, facilitate staff engagement with evidenced-based practice programs, leading to their successful implementation and adoption [32,33,34,38,39,40,41,42,43,44,48,53,54,61]*.

Successful QCP-M implementation and adoption is facilitated where staff feel involved and engaged [32,40,41,53,61,62], where clinical sites have education and training functions to support staff learning [32,33,39,41,42,44] with dedicated champions/implementation leads to support them [38] [32,33,34,38,40,42,43,48,53,54,59,61,62].

#### 3.4.6. Programme Theory 6: Patient Needs

Program theory 6 is supported by three CMOc extracted from twelve papers and is associated with patients’ needs, the perceived benefits of quality interventions for them, and their involvement in intervention design.

*Clinical sites that work in partnership with their patients and their families have an awareness of their needs and understand the benefits of quality initiatives for them [32,34,37,44,49,51,57,58,63]*.

Where patients and their families are aware of standards of care [32], are involved [49,51,64] work in partnership [49,63] or co-design quality interventions [37,44,49,51,57,65] and where staff have an awareness of the perceived needs of patients [37], patients are empowered, facilitating audits of standards of care.

## 4. Discussion

This paper presents an RRR of published and grey literature relating to the implementation of Quality Care-Process Metrics (QCP-M) to improve the quality of care across nursing and midwifery settings. The review is relevant in the context of broader healthcare debates that recognize the implementation challenges associated with improvement approaches. The Programme Theory (PT) generates a better understanding of ways to promote the successful implementation of QCP-M. In implementing any improvement within healthcare, there is a risk that the implementation methodology fails to consider the social interactions and complex dynamics in healthcare settings [66,67]. The reality is that improvement interventions often fail to achieve widespread uptake despite strong evidence for their benefit [68,69]. This suggests that to improve health services then the study of quality improvement interventions and methodologies in healthcare is of paramount importance [70]. There is also a need for further evidence-based research that can offer practical opportunities to promote transformational improvement across a health system are needed [70,71]. This speaks to locating improvement interventions, such as QCP-M, within conceptual frameworks that emphasize systems thinking and avoids seeing them as decontextualized toolkits [72].

### 4.1. Summary of Findings

The PT findings outline key characteristics that promote the successful implementation of QCP-M in nursing and midwifery practice in a range of contexts. These will be contextualized within the quality improvement and implementation science literature.

Program theory 1 focuses on the evidence base and accessibility of the QCP-M and on their ease of use by nurses and midwives working in busy and complex care environments. This review found nurses and midwives adopt QCP-M more readily when they appreciate the evidence underpinning the metric and can trial and adapt it to their local context. Intervention characteristics, including evidence, adaptability, and trialability, and how they are perceived by those using them, influence implementation [8,73]. The need for ongoing adaptation of an intervention to its context is a crucial recommendation. It is recognized as vital for achieving sustainable change and lasting improvement rather than diminishing outcomes over time [8,74]. Implementation planners should provide education on the intervention and adopt a responsive, iterative implementation approach that enables stakeholders to make intervention adaptations as appropriate (Braithwaite 2018).

The second program theory examines the influence of external factors on QCP-M implementation. It highlights the role of external and internal strategies to support implementation. It identified that successful implementation requires input from external strategies aligned to internal organizational strategies through, for example, education and training, champions, and implementation leads. This is especially effective for well-networked, outward-facing organizations that collaborate readily. This finding supports evidence that external strategies, e.g., national implementation programs and policy initiatives, often termed a “policy push”, generate organizational motivation and intervention spread. However, external strategies alone do not increase the capacity of organizations to implement [8,73]. Managers of healthcare organizations face the unenviable task of working with limited resources (Foshay and Kumziemsky, 2014) to deliver high-quality care. Organizations face pressure to sustain a quality service and deliver change while achieving operational and strategic excellence and keeping the service patient-focused [75]. Therefore, it is essential that internal strategies focus on quality improvement initiatives that align with organizational goals and identify meaningful performance metrics [76]. This finding is practically important so that organizations do not assume that external strategies are sufficient and that internal investment is also required to support implementation.

The third PT relates to existing cultures and systems within clinical sites. Organizational culture, which is an active, interacting facet of implementation [8,77,78,79]. This review is consistent with the literature, which found that a strong learning culture supports staff information needs, fostering staff commitment and readiness for QCP-M adoption [8,80]. Furthermore, an important enabler of improvement deployment in an organization is a top-down and bottom-up approach with support from management and the engagement of teams of clinicians and other staff in a supportive culture [81]. Management is seen to have a supportive role in the implementation of any improvement initiative, facilitating, educating, and empowering teams to apply implementation tools [82], and it is reiterated that improvement methodologies need management support and the provision of staff education and training [83].

The fourth PT relates to nurses’ and midwives’ knowledge and beliefs. Organizational awareness of their beliefs speaks in particular to person-centeredness and the need for person-centred approaches to care, inclusive of every person involved in the patient’s care, not just the patient [84,85]. The importance of knowing both our own, our colleagues, our patients, and our organizations’ values and beliefs [86] is important because clarifying values and beliefs underpins our work and practice and makes clear that improvement initiatives (such as the QCP-M) are much more than decontextualized toolkits [72] and require a consensual decision-making approach.

Clinical sites that recognize the needs and the preferences of the individual nurse and midwife and have respect for them as persons are well placed to deliver expected outcomes from interventions such as the QCP-M. These factors can also be seen as ‘humanizing’ process improvement, which is a key context for any successful improvement process [87]. They also acknowledge the essential requirement of active staff engagement and empowerment in any quality improvement strategy [88], which have been shown to deliver expected outcomes from collaborative, inclusive, and participative improvement interventions [88,89,90] that contribute to overall organizational culture.

The fifth PT builds on the staff theme of PT four, extending the culture of organizational learning, and highlights the meaningful engagement of nurses and midwives in the implementation process as a key characteristic of success. Within the contextual settings of contemporary organizations, it is suggested that there is recognition that individuals’ potential to be ‘maximized and realized’ has resulted in a greater emphasis on finding conditions that enable people to ‘flourish’ in their work environments [91]. Effective staff engagement includes engaging staff in the learning process during implementation. Learning, in this case, is most effective in influencing implementation success when it is integrated into an everyday workflow where staff interact freely with QCP-M implementation leads and champions. This supports the view that improvement initiatives are better in an environment with a nuanced learning system with effective feedback loops. This strategy helps to build momentum for a quality improvement culture [69]. Those planning QCP-M implementation should consider the importance of implementation leads and champions in supporting QCP-M. These leads and champions can play an important role in supporting effective teamwork, having an awareness of staff time and workload management and the relationships among staff, enabling the creation of a democratic and inclusive culture that in itself facilitates space for the creation of person-centred practice [88]. It has been identified that those at middle manager level are most effective for positively influencing healthcare implementation as they diffuse and synthesize information, mediate between strategy and day-to-day activities, and sell innovation implementation [92].

The sixth and final program theory relates to patient needs. The study found that clinical sites that partner with patients and their families to enhance understanding of the rationale for, and the intended outputs of QCP-M, provide evidence of more empowered patients, and this facilitates audits of standards of care. This is further enhanced where patients are involved in or co-design QCP-M for implementation and where there is a shift of focus to more patient-centred care instead of medically dominated and disease-focused [93]. Furthermore, person-centeredness, underpinned by robust philosophical and theoretical concepts, has an increasingly solid footprint in policy and practice [94]. This concurs with quality improvement literature which identifies that patient-centred organizations are more likely to implement quality improvement and change [95,96,97,98,99].

### 4.2. Contribution to the Existing Literature and Application of Findings

To the best of our knowledge, this is the first RRR on the effective implementation of QCP-M in nursing and midwifery. It should facilitate healthcare teams’ learning, contribute to service improvement and inform future implementation planning. The review contributes new knowledge to the nursing and midwifery literature and, more broadly, to quality improvement and implementation science.

This review provides actionable findings that discern positive influences and identify some key implementation strategies required to support the sustainable scaling-up of QCP-Ms. The findings provide guidance to healthcare policymakers and practitioners across the health system embarking on similar quality initiatives.

### 4.3. Strengths and Limitations of This Review

The realist analysis approach embraces contextual complexity providing a more in-depth understanding of why and how things work [18]. It gives an outcomes-focused knowledge synthesis from a content expert panel and published research, ensuring relevance with emergent healthcare priorities [100]. This review was systematic and comprehensive but not exhaustive as the literature search was limited to English language papers, and the studies included in this review, while not deliberately geographically limited, are predominantly from Europe. However, we note that the exclusion of non-English publications from systematic reviews on interventions has been evidenced to have a minimal effect on overall conclusions and can be seen as a viable methodological shortcut, especially for rapid reviews [101]. Methodological rigor was strengthened by adhering to RAMESES II. The reviewers were immersed in seeking explanations and testing theories until data saturation was achieved [102]. However, it must be acknowledged that it is an interpretive analytic process. The CMO configurations identified were not clearly defined in the literature and are therefore theorized and need further testing.

### 4.4. Further Research

This RRR has expanded program theory on QCP-M implementation across nursing and midwifery practice. Future empirical studies to further advance our understanding are advised. Other research should test and refine this program theory using primary data based on the experiences and perspectives of different stakeholders involved with QCP-M across the healthcare system. This study did not thoroughly examine the interrelationship between program theories, and further research could do this. The RRR has identified six PTs and their inherent CMOc relating to the research question. A follow-up Realist Evaluation may further explore and test these PTs and CMOc in a range of real-life contexts of nurses and midwives and provide an opportunity for the PTs to be confirmed, refuted, or refined [103] to further develop a theory of how and why QCP-M work or do not work, in what circumstances and for whom.

## 5. Conclusions

Adopting a RRR, this paper provides an understanding of the complex dynamics of implementing nursing and midwifery QCP-M as a quality improvement initiative to promote safe, quality person-centred care. RRR seeks to inform the researcher’s understanding of the relationships between context, mechanism, and outcomes (CMOc) for specific interventions [104] and enabled us to have an understanding of what about QCP-M implementation worked/didn’t work, for whom, and in what circumstances. This understanding facilitated progression from our IPT to the evidence-based refinement of causative factors [14,105]. It is also suggested that while RRR is a useful research approach, that it should allow for organic interpretation [106]. Our mapping of CMOc to existing implementation theory using CFIR was an example of the organic use of RRR, which, whilst following the RAMESES II guidelines, enabled us to contextualize our findings within implementation science fully. This RRR focused on how those working in complex, busy nursing and midwifery clinical settings feel supported in practical ways to adopt QCP-M into their practice. These results highlight important considerations for implementation planners and policymakers to target strategies that support the process and foster support for and from staff using QCP-M systems. The program theories presented in this paper may help guide implementation planning.

## Figures and Tables

**Table 1:** The inclusion and exclusion criteria applied.

Criterion	Inclusion	Exclusion
Population	Humans	Any study population other than humans; e.g., animal studies
Language	Written in English	Any other language that is not English
Time Period	January 2012–July 2020	Outside this time period
Study Focus	Studies that report on the implementation and/or evaluation of the implementation of QCP-M or other nursing or midwifery quality care measurement processes, both nationally and internationally	Articles that do not look at QCP-M/or other healthcare interventions and initiatives
Type of Study	Peer-reviewed primary studies from academic journals and grey literature from, for example, reference lists and institutional repositories	Non-peer reviewed articles; e.g., newspaper articles, opinion pieces, and reviews
Geographic Location	Any location within an international context	None

**Table 2:** PICO search terms used in the review of the literature.

Question	PICO	Search Terms
What factors enable the successful implementation of a suite of Quality Care Nursing and Midwifery Metrics in nursing and midwifery practice?	P I O	‘Nurse’ OR ‘Midwife’ OR ‘nurse specialist’ OR ‘nurse practitioners’ OR ‘clinical nurse specialists’ OR ‘midwife specialist’ AND ‘quality care’ OR ‘clinical care’ ‘nursing care’ and ‘measurement’ and ‘processes’ and ‘indicators’ as separate terms AND ‘facilitators’ OR ‘enablers’ OR ‘implementation’

## Data Availability

The data presented in this study are available as part of the article and also included within the Supplemental Files.

## Supplementary Materials

The following are available online at https://www.mdpi.com/article/10.3390/ijerph182211932/s1, File S1: Representation List of Reference & Expert Panels, Table S2: Extraction Template with CFIR Framework, Table S3: Included Documents and Table S4: CMOc and PT Refinement.
