# Supplementary material for: A Rapid Realist Review of Quality Care Process Metrics Implementation in Nursing and Midwifery Practice
Source: Int J Environ Res Public Health. 2021 Nov 13;18(22):11932. doi: 10.3390/ijerph182211932 (PMC8621300; doi:10.3390/ijerph182211932)
Supplement: Supplementary file 1 [file ijerph-18-11932-s001.zip › 1.Supplementary File S1_Reference & Expert Panels.pdf]

Supplementary File 1

Representation List of Reference Panel

- Professor Laserina O'Connor, Professor of Clinical Nursing, Director of the Professional Certificate Pain Management, Diploma Pain Management & MSc Advanced Pain Management/Prescriptive Authority, Director Graduate Diploma Chronic Illness, Director CPD Evidence Based Practice & Research Champions and Principal Investigator, UCD School of Nursing Midwifery and Health Systems
- Dr. Anne Gallen, Interim Area Director, HSE Nursing & Midwifery Planning & Development Dublin Mid Leinster and National Lead for Nursing & Midwifery Quality Care-Metrics
- Associate Professor Mary Casey, Lecturer, UCD School of Nursing Midwifery and Health Systems
- Dr. Denise O'Brien, Assistant Professor/Lecturer in Midwifery, Programme Director MSc Advanced Practice Midwifery, Affiliated Investigator UCD Perinatal Research Centre and Member of the Advisory Committee UCD Sexual Exploitation Research, UCD School of Nursing Midwifery and Health Systems
- Dr. Rita Smith, Assistant Professor and Lecturer, UCD School of Nursing Midwifery and Health Systems
- Ms. Carmel Bradshaw, Lecturer and Course Director, Department of Nursing and Midwifery, University of Limerick
- Dr. Maria Noonan, Lecturer, Department of Nursing and Midwifery, University of Limerick
- Dr. Owen Doody, Senior Lecturer, Department of Nursing and Midwifery, University of Limerick
- Dr. Yvonne Corcoran, Assistant Professor, School of Nursing, Psychotherapy and Community Health, Dublin City University
- Dr. Sylvia Murphy Tighe, Lecturer in Midwifery, Department of Nursing and Midwifery, University of Limerick
- Ms. Rosemary Lyons, Lecturer, Department of Nursing and Midwifery, University of Limerick
- Ms. Liz Dore, Librarian, Education and Health Sciences, Library and Information Services Division, University of Limerick
- Ms. Jane O'Doherty, Research Assistant, Department of Nursing and Midwifery, University of Limerick

- Ms. Máire McGeehan, Research Assistant, Department of Nursing and Midwifery, University of Limerick

Representation List of Expert Panel

- Dr. Seán Paul Teeling, Programme Director for the Professional Certificate and Graduate Diploma in Process Improvement, Lecturer and Assistant Professor Health Systems, UCD School of Nursing Midwifery and Health Systems
- Professor Martin McNamara, Professor, Associate Dean and Programme Director for the MSc in Leadership, Innovation and Management for Healthcare and related programmes in leading, innovation and managing in health systems – and for the MSc and Graduate Certificate in Health Professions Education, UCD School of Nursing Midwifery and Health Systems
- Professor Alice Coffey, Professor of Nursing at Department of Nursing and Midwifery and Lead of the HRI Health Implementation Science Cluster (HIST), Department of Nursing & Midwifery, Faculty of Education and Health Sciences, University of Limerick
- Professor Veronica Lambert, Full Professor of Children and Family Nursing/Health at Dublin City University, School of Nursing, Psychotherapy and Community Health
- Dr. Carmel Davies, Associate Dean of Teaching and Learning and Lecturer, UCD School of Nursing Midwifery and Health Systems
- Dr. Timothy Frawley, Programme Director Higher Diploma in Mental Health Nursing, Associate Dean for Taught Graduate Studies, Head of Subject – Mental Health Nursing and Assistant Professor, UCD School of Nursing Midwifery and Health Systems
- Dr. Catherine Redmond, Lecturer and Assistant Professor, UCD School of Nursing Midwifery and Health Systems
- Dr. Suja Somanadhan, Head of Subject for Children's Nursing, programme Director Graduate Diploma in Emergency Nursing (Children), Assistant Professor in Children's Nursing, Fulbright Scholar 2019-2020, UCD Centre for Interdisciplinary Research, Education and Innovation in Health Systems (UCD IRIS) and Lecturer, UCD School of Nursing Midwifery and Health Systems
- Dr. Dympna Tuohy, Course Director Postgraduate Programmes and Lecturer, Department of Nursing & Midwifery, Faculty of Education and Health Sciences, University of Limerick
- Ms. Marlize Barnard, Research Assistant, PhD Candidate and Occasional Lecturer, UCD School of Nursing Midwifery and Health Systems
