# Supplementary material for: A Rapid Realist Review of Quality Care Process Metrics Implementation in Nursing and Midwifery Practice
Source: Int J Environ Res Public Health. 2021 Nov 13;18(22):11932. doi: 10.3390/ijerph182211932 (PMC8621300; doi:10.3390/ijerph182211932)
Supplement: Supplementary file 1 [file ijerph-18-11932-s001.zip › 2.Supplementary Table S2 Extraction Template with CFIR Framework.pdf]

Supplemental File S2

Table S2. Extraction Template with CFIR Framework included

CFIR Constructs	Included Papers	Context	Mechanism	Outcome
**Outer setting**	Paper 1 Cheyne et al.(2013) Midwifery programme	Support for programme at national and local level	facilitated by the presence of resources and local champions	enabled midwifes to focus on implementation
**Inner setting**	Paper 1 Cheyne et al.(2013) Midwifery programme	Programme was integrated with existing local study site protocols	and negotiated for local use with the multidisciplinary team	leading to standardised practice, midwifery led care and adherence to principles
**Inner setting**	Paper 1 Cheyne et al.(2013) Midwifery programme	Good midwifery practice existed within the study sites	so the intervention was adapted to compliment local midwifery practice	resulting in a perceived increase in efforts towards normality and reduction in intervention rates
**Process**	Paper 1 Cheyne et al.(2013) Midwifery programme	Cohesive message on standards and practice between study sites	was supported by new pathways to standardise and structure practice geographically	resulting in a perception that communication between geographical units had improved and that protocols were implemented locally
**Outer setting/individuals**	Paper 1 Cheyne et al.(2013) Midwifery programme	Wider implementation wasn't considered in all sites (example, GPs in certain sites)	so there were no mechanisms to engage with GPs	which resulted in GPs feeling alienated and excluded
**Inner setting**	Paper 1 Cheyne et al.(2013) Midwifery programme	An existing practice culture	resulted in pathways being used heuristically	that resulted in obstetricians being supportive and midwifes perceived as equals
**Inner setting**	Paper 2 Francis Coad (2018) Falls prevention programme	Organisational culture of management support	prioritised staff participation in the falls prevention programme	enabling the implementation of evidenced based falls prevention strategies
**Inner setting**	Paper 2 Francis Coad (2018) Falls prevention programme	Existing communication structures	used a feedback loop to report on the actions of programme implementation	which triggered proactive behaviour and focused attention on the falls prevention programme

**Inner setting**	Paper 2 Francis Coad (2018) Falls prevention programme	Existing workloads	hampered staff engagement with the falls prevention programme	meaning staff did not participate in the programme
**Inner setting**	Paper 2 Francis Coad (2018) Falls prevention programme	Use of a community of practice	brought together likeminded people	enabling the implementation of evidenced based falls prevention strategies
**Process**	Paper 2 Francis Coad (2018) Falls prevention programme	Senior management investment in giving staff time	motivated staff to participate	enabling the implementation of evidenced based falls prevention strategies
**Process**	Paper 2 Francis Coad (2018) Falls prevention programme	Staff receptivity to falls prevention programme	was facilitated by available education and training, feedback loop, knowledge sharing and social learning opportunities	enabling the implementation of evidenced based falls prevention strategies
**Process**	Paper 2 Francis Coad (2018) Falls prevention programme	Knowledge deficit on the full potential of falls prevention programme	was addressed by available education and training, feedback loop, knowledge sharing and social learning opportunities	enabling the implementation of evidenced based falls prevention strategies
**Individuals**	Paper 2 Francis Coad (2018) Falls prevention programme	Community of Practice member characteristics	provided a high level of local knowledge in falls prevention	enabling the implementation of evidenced based falls prevention strategies
**Inner setting**	Paper 3 Dewi Staplers (2017) Nurse Sensitive Outcomes	Busy work environment/workload was obviated by	incorporating the Nurse sensitive outcomes (NSO) monitoring into nurses' regular duties and responsibilities	so that NSOs were not seen as additional work and burden
**Process**	Paper 3 Dewi Staplers (2017) Nurse Sensitive Outcomes	Clarity of local and national policies was facilitated by	education and training on NSOs for staff	resulting in nurses' understanding the implications of data for benchmarking and use nationally to inform patient outcomes
**Process**	Paper 3 Dewi Staplers (2017) Nurse Sensitive Outcomes	Cohesive message on standards and practice between study	education and training on NSOs for staff	so that NSOs were not seen as additional work and burden

		sites was facilitated by		
**Individuals**	Paper 3 Dewi Staplers (2017) Nurse Sensitive Outcomes	Practice environment supported by	practitioner autonomy and feedback loops	leading to satisfaction with work environment
**Process**	Paper 3 Dewi Staplers (2017) Nurse Sensitive Outcomes	Knowledge deficit on the full potential of NSO data was addressed by	education and training on NSOs for staff	resulting in nurses' understanding the implications of data for benchmarking and use nationally to inform patient outcomes
**Inner setting**	Paper 4 Sims (2018) Intentional Rounding	Busy work environment/workload was obviated by	staff who engaged with programme as they saw the benefits for patient care and family communication	which led to increased staff vigilance and awareness of psychological and physical patient needs
**Inner setting**	Paper 4 Sims (2018) Intentional Rounding	Other competing initiatives were obviated by	staff who engaged with programme as they saw the benefits for patient care and family communication	enabling workload organisation for nursing staff and their ability to anticipate person requirements
**Process**	Paper 4 Sims (2018) Intentional Rounding	Availability of staff education and training	facilitated awareness of physical and psychological patient care needs	enabling ward managers to audit the standard of care
**Intervention characteristics**	Paper 4 Sims (2018) Intentional Rounding	Deployment - staged or system wide affected	how/when staff engaged with the programme	enabling staff to use the programme to facilitate improvement in patient outcomes
**Inner setting**	Paper 4 Sims (2018) Intentional Rounding	Existing practice culture (embedded) facilitated	provision of time for intentional rounding	until it became 'business as usual'/standard practice
**Process**	Paper 4 Sims (2018) Intentional Rounding	Senior management buy in and support for programme facilitated	support from stakeholders	resulting in adherence to principles
**Process**	Paper 4 Sims (2018) Intentional Rounding	Availability of resources to carry out rounds	such as intentional rounding documentation/record was used by staff	Enabling ward managers to audit the standard of care and the organisation to note increased quality of care
**Individuals**	Paper 4 Sims (2018) Intentional	Patients aware of standards of	empowered patients to nurses	Patients have expectation of

	Rounding	care	visibility of care	care and quality of interaction Patient empowerment Visibility of nursing work is clear to all involved in the process of care
**Process**	Paper 4 Sims (2018) Intentional Rounding	Champion and Buddy system in the organisation	meant nurses engaged with the buddies	Increasing their engagement with the intentional rounding
**Inner setting**	Paper 4 Sims (2018) Intentional Rounding	Work environment/workload was organised to	facilitate engagement with patients and their families	reducing risks such as falls, pressure ulcers - improved patient psychological and physical outcomes
**Intervention characteristics**	Paper 5 Cross and Cheyne (2018) Maternity Service	The use of a women's hand held record across maternity services	emphasised a framework of common assessment, that with training and follow through	might be seen to address the variance in skills
**Outer setting**	Paper 5 Cross and Cheyne (2018) Maternity Service	Different stages of training and implementation across healthboards	led to correspondingly different approaches to patient assessment by Midwifes	that resulted in the midwives experiential learning being a key factor in motivating longer-term implementation.
**Inner setting**	Paper 5 Cross and Cheyne (2018) Maternity Service	Large Midwifery caseloads and associated time pressure	led to correspondingly different approaches to patient assessment by Midwifes	that resulted in Midwifes not always following Evidence Based Practice (EBP)
**Outer setting**	Paper 5 Cross and Cheyne (2018) Maternity Service	Caseload variance from healthboard to healthboard	required joint working across the healthboards to develop materials for programme implementation	which meant variation in implementation lead to consequential difference in Midwifes' experience of EBP
**Outer setting**	Paper 5 Cross and Cheyne (2018) Maternity Service	An International evidence-based approach	promoted empathetic partnerships; progressive individual feedback on performance and personal follow-up coaching by champions	that led to training and support being embedded in practice
**Inner setting**	Paper 5 Cross and Cheyne (2018) Maternity Service	An integrated policy	enabled staff to use standardised guidance	leading to Midwifes' adoption of the accessible practice model

			documents	
**Process**	Paper 5 Cross and Cheyne (2018) Maternity Service	Coordination of the implementation of policy	was supported by funded learning materials and change champions	that when present led to advanced training and implementation, and when not present lead to a gap in the capacity to develop strength-based skills
**Intervention characteristics**	Paper 5 Cross and Cheyne (2018) Maternity Service	Introduction of the policy tools to service users	was supported by gradual rather than abrupt intervention	that developed Midwifes' motivation to change and promoted equality in care provision
**Intervention characteristics**	Paper 5 Cross and Cheyne (2018) Maternity Service	Evidence-based midwifery practices	were supported by use of standarised records, care pathways and risk assessments	leading to some patients' (women's) active engagement; however the degree of engagement was influenced by socio economic factors of patients.
**Resources**	Paper 5 Cross and Cheyne (2018) Maternity Service	Variance in access to Information Technology	was offset by the prior learning and experience of midwifes	but the variation in access to Information Technology led to a consequential difference in staff experience of EBP
**Process**	Paper 6 Williams et al. (2016) IPC	Facilitators/Champions facilitated and supported clinical practice	which facilitated staff engagement and adherence with infection control standards in practice	leading to adherence with best practice in infection prevention
**Process**	Paper 6 Williams et al. (2016) IPC	Facilitators/Champions facilitated and supported clinical practice	enabling clinical staff to feel supported in their practice	resulting in increased compliance with Infection control protocols, feelings of collegiality and motivated staff
**Intervention characteristics**	Paper 6 Williams et al. (2016) IPC	The intervention (IC) was seen as a high priority for the organisation with an emphasis placed on facilitation	motivating staff to reflect on their practice	leading to good individual habitual behaviours

**Process**	Paper 6 Williams et al. (2016) IPC	Facilitators/champions provided practice based education for clinical staff to counteract lack of priority or time for formal training	meaning staff were consistently reminded of the sense of relevance to their own practice	leading to a heightened awareness of infection prevention in clinical areas
**Process**	Paper 7 Avra et al. (2018) Health care for adults with intellectual and developmental disabilities	Facilitators/Champions were present	who staff engaged with	resulting in staff adopting and embedding new practices
**Inner setting**	Paper 7 Avra et al. (2018) Health care for adults with intellectual and developmental disabilities	Passive leadership endorsement	discouraged staff engagement	leading to variability in adoption of new practices
**Inner setting**	Paper 7 Avra et al. (2018) Health care for adults with intellectual and developmental disabilities	Low morale and high staff turnover	discouraged staff engagement	leading to variability in adoption of new practices
**Inner setting**	Paper 7 Avra et al. (2018) Health care for adults with intellectual and developmental disabilities	High workload and competing demands	discouraged staff engagement	resulting in lack of engagement with new practices
**Outer setting**	Paper 7 Avra et al. (2018) Health care for adults with intellectual and developmental disabilities	Where the QI strategy was supported by the National Implementation Research Network	staff engaged with provided resources (e.g. implementation logs/champions/electronic reminders and prompts)	resulting in increased chance of intervention success
**Inner setting**	Paper 7 Avra et al. (2018) Health care for adults with intellectual and developmental disabilities	Variability in available resources	led to variability in staff engagement with the intervention	leading to corresponding variation in degree of engagement with intervention
**Inner setting**	Paper 7 Avra et al. (2018) Health care for adults with intellectual and developmental disabilities	Competing initiatives	meant staff had limited time to engage with the intervention	leading to lack of or passive engagement with intervention

**Individuals**	Paper 7 Avra et al. (2018) Health care for adults with intellectual and developmental disabilities	Patient caseload	meant there was a patient population that staff were often unfamiliar with	leading to staff concerns about ability to care for the patient demographic
**Intervention characteristics**	Paper 8 Bunn et al. (2017) Diabetes in PLWD	Positive attitudes towards PLWDD	meant staff felt that PLWDD have the potential to be involved in self- management	resulting in staff supporting and engaging in self management by PLWDD and their carers
**Culture**	Paper 8 Bunn et al. (2017) Diabetes in PLWD	Person-centred approaches to care planning	generated trust between family and HCPs	leading to a better fit between care planning and patient and carer needs and (potentially) a lessening of the burden of medicalisation experienced by PLWDD and their families
**Individuals**	Paper 8 Bunn et al. (2017) Diabetes in PLWD	HCPs were expected to develop skills that enhanced the delivery of individualised and tailored care to PLWDD	that created an expectation in patients and HCPs that the management of diabetes for PLWD was important	leading to better engagement in self-management by PLWDD and family carers
**Culture**	Paper 8 Bunn et al. (2017) Diabetes in PLWD	Family carers were routinely involved in care planning and information sharing	meaning they believed their contribution was recognised and appreciated	leading to development of effective self-management strategies on the part of the family & carers
**Intervention characteristics**	Paper 8 Bunn et al. (2017) Diabetes in PLWD	Assistive technology needed to be tailored and adapted to the needs and requirements of PLWDD and family	PLWDD and family engaged with and used assistive technology in their management of diabetes and dementia	resulting in more effective and sustained use of assistive technology to maintain autonomy
**Culture**	Paper 9 Gee et al.(2017) Recovery oriented training in MH rehab	Collaborative action planning between staff groups and service users	meant staff felt engaged, valued and involved	resulting in a sustainable change of practice
**Intervention characteristics**	Paper 9 Gee et al.(2017) Recovery oriented training in MH rehab	Incorporating recovery into an existing change programme	facilitated staff engagement, enthusiasm and change ‘receptiveness’	resulting in a sustainable change of practice

**Inner setting**	Paper 9 Gee et al.(2017) Recovery oriented training in MH rehab	Job uncertainty and fear	hindered staff engagement, enthusiasm and change 'receptiveness'	resulting in a lack of sustainable change of practice
**Process**	Paper 9 Gee et al.(2017) Recovery oriented training in MH rehab	Regular collaborative meetings	helped staff members feel supported by their peers and managers in the change programme	resulting in a sustainable change of practice
**Process**	Paper 9 Gee et al.(2017) Recovery oriented training in MH rehab	Change agents or champions	encouraged other staffs' engagement, enthusiasm and change 'receptiveness'	resulting in a sustainable change of practice
**Process**	Paper 9 Gee et al.(2017) Recovery oriented training in MH rehab	Management support and support of role flexibility	helped staff members feel supported by their peers and managers in the change programme	resulting in a sustainable change of practice
**Inner setting**	Paper 9 Gee et al.(2017) Recovery oriented training in MH rehab	Modifying organisational structures to support change	helped staff members feel supported by their peers and managers in the change programme	resulting in a sustainable change of practice
**Individuals**	Paper 10 Brown et al.(2018) Care planning for patients with long term conditions	People(patients) had an understanding of their role in managing their own condition	that led them to reflect on their current health status and ask questions	resulting in engagement of patient in consultation
**Culture**	Paper 10 Brown et al.(2018) Care planning for patients with long term conditions	Partnership working (patient and practitioner)	meant practitioners had time to spend discussing what is important to the patient	leading to patients taking ownership of their health outcomes
**Individuals**	Paper 10 Brown et al.(2018) Care planning for patients with long term conditions	Patients wanting to improve aspects of their health via support from practitioner	enabled clinical staff to feel supported in their practice	leading to meaningful and achievable goals being set
**Individuals**	Paper 10 Brown et al.(2018) Care planning for patients with long term conditions	Patients understanding how their behaviours and lifestyle affect their condition	enabled them to have ownership of their own care	resulting in them making healthier lifestyle choices improving their health outcomes
**Culture**	Paper 10 Brown et al.(2018) Care planning for patients with	An environment that facilitated and motivated	enabled practitioners to support patients	meaning patients engaged in shared decision making and

	long term conditions	knowledge exchange		self-management
**Individuals**	Paper 10 Brown et al.(2018) Care planning for patients with long term conditions	Patients were proactive in their care	which enabled practitioners to support them	resulting in their making healthier lifestyle choices improving their health outcomes
**Culture**	Paper 10 Brown et al.(2018) Care planning for patients with long term conditions	Relationships of trust and respect between patient and practitioner	enabled practitioners to support patients	resulting in shared decision making
**Outer setting**	Paper 10 Brown et al.(2018) Care planning for patients with long term conditions	Patients required holistic, personalised support	which enabled them to feel equipped with knowledge and skills	so that they were confident in managing their own health and health outcomes
**Inner setting**	Paper 11 Li et al. (2018)The implementation of evidence based factors	Organisational openness to trialling new ideas and learning	meant staff engaged with new evidence based practice (EBP)	increasing the success of new EBP implementation
**Culture**	Paper 11 Li et al. (2018)The implementation of evidence based factors	Culture of some grades of HC staff having too much autonomy	meant staff were resistant to new EBP	leading to failure of new EBP implementation
**Inner setting**	Paper 11 Li et al. (2018)The implementation of evidence based factors	Transformational leadership supportive of staff	facilitated staff self-perception as part of the implementaiton team	leading to a learning culture and sustainable change
**Inner setting**	Paper 11 Li et al. (2018)The implementation of evidence based factors	Leadership that was unsupportive of change or willing to hold staff accountable for change	meant staff felt unmotivated to change	leading to failure of new EBP implementation
**Inner setting**	Paper 11 Li et al. (2018)The implementation of evidence based factors	Variation in availability of financial resources across different departments and sites	meant variation in staff access to EBP supports	resulting in staff struggling to provide adequate service
**Inner setting**	Paper 11 Li et al. (2018)The implementation of evidence based factors	Staffing and workload demands	meant staff were less likely to engage with change	leading to failure of new EBP implementation
**Inner setting**	Paper 11 Li et al. (2018)The implementation of evidence based factors	Time and competing demands	meant staff were unable to engage with or failed to prioritise new EBP	leading to failure of new EBP implementation

**Process**	Paper 11 Li et al. (2018)The implementation of evidence based factors	Lack of provision of education and training	did not facilitate staff engagement	leading to failure of new EBP implementation
**Process**	Paper 11 Li et al. (2018)The implementation of evidence based factors	Where feedback mechanisms were present	they encouraged staff engagement	resulting in higher rates of EBP learning implementation and increased likelihood of EBP sustainability over time
**Process**	Paper 11 Li et al. (2018)The implementation of evidence based factors	Organisation had Champion role for new EBP innovations	meaning staff engaged with Champions who were 'on the floor', had expertise and were familiar to them	increasing the success of new EBP implementation
**Intervention characteristics**	Paper 12 Bryce et al. (2018) Implementing change in primary care practice	A complex change initiative and demanding work environment	required a champion or champions to drive the service development and implementation	without which sites struggled to adapt and evolve in response to changing demands on the service
**Outer setting**	Paper 12 Bryce et al. (2018) Implementing change in primary care practice	Patients were uninformed	as the service had not been developed with, or advertised to, local residents prior to its introduction.	leading to resistance to change from patients and, to a lesser extent, their families.
**Inner setting**	Paper 12 Bryce et al. (2018) Implementing change in primary care practice	Poor communication to staff	meant felt unclear about where responsibility ended, both in terms of accountability for clinical decisions, and in defining the limits of care	resulting in ongoing uncertainty that limited further implementation
**Outer setting/individuals**	Paper 12 Bryce et al. (2018) Implementing change in primary care practice	Focus on an outcome of reducing unplanned admissions	clashed with staffs' professional values (supporting person-centred care) and patient-focused outcomes	leading to uncertainty over boundaries of care and creating tension and burden for staff.
**Inner setting**	Paper 12 Bryce et al. (2018) Implementing change in primary care practice	Development of a service model to suit local needs	with no local supports in place for staff to engage with	leading to significant levels of sickness/ absence in some teams (but correlation rather than causation established).
**Process**	Paper 12 Bryce et al. (2018)	Initial support from	with no local supports in place	leading to significant levels of

	Implementing change in primary care practice	commissioners of the PACT service was short lived	for staff to engage with	sickness/ absence in some teams (but correlation rather than causation established).
**Process**	Paper 12 Bryce et al. (2018) Implementing change in primary care practice	Context of developing professional expertise derived from critical reflection on implementation of evidence-informed practice	but staff lacked the training and/or confidence in critically assessing the value of this knowledge	resulting in staff potentially undervaluing their individual and collective learning from implementation
**Inner setting**	Paper 13 Pearson et al. (2015) Collaborative care in offender mental health	There was an infrastructure to support practitioners work	that gave practitioners the opportunity to develop their skills and have their practice supported	resulting in monitoring of individuals in need of care, and the provision of feedback and support that facilitated practitioners' skill development
**Outer setting**	Paper 13 Pearson et al. (2015) Collaborative care in offender mental health	Congruence at both strategic and practitioner levels	that took account of practitioners values and goals	led to efficient implementation were present, and piecemeal implementation were absent
**Process**	Paper 13 Pearson et al. (2015) Collaborative care in offender mental health	Facilitative organisational measures to support intervention development.	enabled practitioners to understand their role, responsibilities, and the contribution that they make within a system of care	leading to collaborative practice
**Intervention characteristics**	Paper 13 Pearson et al. (2015) Collaborative care in offender mental health	Communication systems (ICT) and inter-organisational agreements facilitated information sharing and care planning	as practitioners knew who to contact and how and believed that their referrals would be welcomed	providing an opportunity for relationship building
**Individuals**	Paper 13 Pearson et al. (2015) Collaborative care in offender mental health	Interventions took place at different levels - individual, team and organisation	but there was a significant lack of concordance between practitioners' and the organisation's perceptions about roles and responsibilities	impacting negatively on practitioner engagement
**Individuals**	Paper 13 Pearson et al. (2015) Collaborative care in offender	Interventions take place at different levels - individual,	and there was concordance between practitioners' and the	meaning practitioners felt that they have contributed

	mental health	team and organisation.	organisation's perceptions about roles and responsibilities	substantively to the development of, and had an ongoing part to play in the implementation of, the proposed service changes
**Individuals**	Paper 13 Pearson et al. (2015) Collaborative care in offender mental health	Practitioners had an understanding of, and skills in mental health care	facilitated by their reflection on their own practice	leading to the examination of assumptions that underpin practise that could inform practitioners' relationship-building
**Individuals**	Paper 14 Yamada et al. (2018) Barriers and enablers to paediatric asthma management	Staff were knowledgeable in use of asthma guidelines	but some staff used them and some did not	which informed the optimization of the PCAPP prior to its evaluation
**Culture**	Paper 14 Yamada et al. (2018) Barriers and enablers to paediatric asthma management	Social and professional identity	facilitated use of a multidisciplinary approach	which informed the optimization of the PCAPP prior to its evaluation
**Culture**	Paper 14 Yamada et al. (2018) Barriers and enablers to paediatric asthma management	Social influences	affected staffs approach to PCAPP	which informed the optimization of the PCAPP prior to its evaluation
**Culture**	Paper 14 Yamada et al. (2018) Barriers and enablers to paediatric asthma management	Social influences	affected staff concerns over patient use of PCAPP	which informed the optimization of the PCAPP prior to its evaluation
**Outer setting**	Paper 14 Yamada et al. (2018) Barriers and enablers to paediatric asthma management	Parental knowledge and understanding	were supported by education but the education was affected by logistics and time constraints	leading to parental uncertainty due to lack of clarify regarding long-term effects of medication
**Inner setting**	Paper 14 Yamada et al. (2018) Barriers and enablers to paediatric asthma management	Availability of resources (HCP colleagues/Medication costs)	influenced HCP prescribing practices	which informed the optimization of the PCAPP prior to its evaluation
**Inner setting**	Paper 14 Yamada et al. (2018) Barriers and enablers to paediatric asthma management	Availability of resources (EMR)	influenced HCP prescribing practices	which informed the optimization of the PCAPP prior to its evaluation
**Inner setting**	Paper 15 Baatiema et al. (2017)	The level of institutional	how staff engaged with EBP	that informed the quality of the

	Barriers and enablers to EBP for acute stroke care	support impacted		staff and patient experience
**Inner setting**	Paper 15 Baatiema et al. (2017) Barriers and enablers to EBP for acute stroke care	Staffing and workload demands impacted on	how staff engaged with EBP	that informed the quality of the staff and patient experience
**Inner setting**	Paper 15 Baatiema et al. (2017) Barriers and enablers to EBP for acute stroke care	Opportunities for professional development impacted on	how staff engaged with EBP	that informed the quality of the staff and patient experience
**Individuals**	Paper 15 Baatiema et al. (2017) Barriers and enablers to EBP for acute stroke care	Variance in individual HCP level of knowledge	hampered HCP uptake of EBP in their everyday practice	that informed the quality of the staff and patient experience
**Inner setting**	Paper 15 Baatiema et al. (2017) Barriers and enablers to EBP for acute stroke care	Resources - space, time, access to beds	hampered HCP uptake of EBP in their everyday practice	that informed the quality of the staff and patient experience
**Inner setting**	Paper 15 Baatiema et al. (2017) Barriers and enablers to EBP for acute stroke care	Poor communication amongst colleagues	hampered HCP uptake of EBP in their everyday practice	that informed the quality of the staff and patient experience
**Inner setting**	Paper 15 Baatiema et al. (2017) Barriers and enablers to EBP for acute stroke care	Lack of clinical leadership	hampered HCP uptake of EBP in their everyday practice	that informed the quality of the staff and patient experience
**Individuals**	Paper 16 McCluskey et al. (2013)	Adopting a new intervention into clinical practice	is influenced by a clinicians' perceptions of their capacity and self-efficacy using an intervention	influences the implementation of evidence based practice
**Individuals**	Paper 16 McCluskey et al. (2013)	Adopting a new intervention into clinical practice	is influenced by clinicians' perceptions of patient capabilities to engage in an intervention	influences the adoption of evidence-based interventions
**Individuals**	Paper 16 McCluskey et al. (2013)	Adopting a new intervention into clinical practice	is influenced by Clinicians' belief of the consequences of using evidence-based practice interventions	impacts on their behaviour to adopt a therapy for patient care
**Process**	Paper 16 McCluskey et al.	.Health services that utilise	prompt clinician attention	and impact on the delivery of

	(2013)	prompts and documentary recording systems		Evidence based practice
**Individuals**	Paper 16 McCluskey et al. (2013)	Clinicians' knowledge and skills related to an intervention	can enable	adoption of Evidence based practice
**Outer setting**	Paper 16 McCluskey et al. (2013)	Health services that commit to invest in resources	enablers clinicians	to deliver EBP
**Inner setting**	Paper 16 McCluskey et al. (2013)	Fostering a social environment that prioritises an intervention as a standing item of business	improves attention to practice	and fosters action in practice
**Culture**	Paper 16 McCluskey et al. (2013)	Organisations that foster conscious raising culture of barriers to an EBP	cultivates a social environment that fosters solutions	to address implementation barriers
**Culture**	Paper 17 Foster et al. (2018)	Organisations that use intervention data for clinical purposes (patient care) rather than clinical performance	influences clinician behaviours	and lead to better adoption of the intervention
**Culture**	Paper 17 Foster et al. (2018)	Organisations that engage stakeholders in intervention design	develop interventions that align with organisation context	and facilitate implementation
**Process**	Paper 17 Foster et al. (2018)	Formally appointed implementation leads	fosters positive support and a responsive environment (feedback)	that fosters organisational implementation
**Process**	Paper 17 Foster et al. (2018)	Organisations that invest time and resources in designing the intervention and strategy for implementation	fosters a social environment readiness	and leads to better implementation success
**Process**	Paper 17 Foster et al. (2018)	Organisations that invest in preparing an organisation and its staff	fosters better readiness for implementation	and leads to better implementation success
**Resources**	Paper 17 Foster et al. (2018)	Organisations that invest in systems support (admin, support, electronic system)	foster commitment	that supports implementation

**Culture**	Paper 18 McHugh et al.(2018)	Organisations that involve the perspectives of clinician's needs and resources	foster better clinician commitment	and lead to better implementation
**Culture**	Paper 18 McHugh et al.(2018)	Support and facilitation that is context sensitive	meets the needs of an organisation	and influence on implementation success
**Inner setting**	Paper 19 Hansen et al.(2017)	Organisations that invest in environmental resources	foster better readiness to implement change	leading to more successful implementation
**Inner setting**	Paper 19 Hansen et al.(2017)	Organisations that invest in system supports and resources	better support clinicians	in the implementation of an intervention
**Inner setting**	Paper 19 Hansen et al.(2017)	Excessive staff workload	fosters lack of attention on intervention	and leads to loss of intervention fidelity
**Inner setting**	Paper 19 Hansen et al.(2017)	Competing initiatives	fosters feelings of confusion	leading to an organisational barrier to implementation
**Individuals**	Paper 19 Hansen et al.(2017)	Staff that are acceptable of new roles and care tasks	enables intervention adoption	and better evidence based practice
**Networks and communications**	Paper 19 Hansen et al.(2017)	Interdisciplinary teams that have communication mechanisms	foster better collaboration and commitment	to implementing an intervention
**Process**	Paper 19 Hansen et al.(2017)	Implementation leads	assist in identification of clear roles and champion the intervention	fosters change efficacy
**Culture**	Paper 19 Hansen et al.(2017)	Staff relations based on hierarchy	fosters negative power relations	that hinder change commitment within an organisation
**Outer setting**	Paper 20 Bee et al. (2015) Barriers and facilitators to service user-led care planning	Divergent views on what services user (SU) and staff (S)want from health care delivery	such as wanting involvement in own care (SU)and being diagnosis & health outcomes focused (S)	results in lack of stakeholder consensus and thus implementation failure
**Outer setting**	Paper 20 Bee et al. (2015) Barriers and facilitators to service user-led care planning	Lack of meaningful engagement with service user/ carers by health care professionals	with little 'relational' interaction with service user	leads to the lack of satisfaction and involvement by service user

**Inner setting**	Paper 20 Bee et al. (2015) Barriers and facilitators to service user-led care planning	Service users that have been trained in participatory decision making	work collaboratively and effectively with healthcare professionals	leading to better service development process
**Culture**	Paper 20 Bee et al. (2015) Barriers and facilitators to service user-led care planning	High staff morale	fosters good partnership	leading to meaningful user involvement in delivery of healthcare
**Process**	Paper 20 Bee et al. (2015) Barriers and facilitators to service user-led care planning	Organisations that enable meaningful service user engagement	improves staff attitude and increases service user esteem	enabling the development of a more responsive health service
**Process**	Paper 20 Bee et al. (2015) Barriers and facilitators to service user-led care planning	Top down approach to intervention implementation	with little collaboration/involvement of stakeholders	results in implementation difficulties
**Process**	Paper 20 Bee et al. (2015) Barriers and facilitators to service user-led care planning	A focus on one setting (inpatient) over another (community)	limits relevance	leads to uneven adoption and only partial implementation of the intervention
**Inner setting**	Paper 20 Bee et al. (2015) Barriers and facilitators to service user-led care planning	Inconsistent information and knowledge gaps regarding mental health care model planning and implementation	does not facilitate full engagement in the care-planning process	results in less adoption and fidelity to the model
**Inner setting**	Paper 22 Bull et al. (2019) Feasibility of the behaviour change wheel to support clinical teams implementing new models of care	Effective and engaged leadership as evidenced by	role modelling, supporting, communicating the 'message' and being acceptable to stakeholders	leads to greater uptake of initiative
**Inner setting**	Paper 22 Bull et al. (2019) Feasibility of the behaviour change wheel to support clinical teams implementing new models of care	Negative impact of organisation's efforts to meet implementation strategy targets is	Staff feeling watched , under scrutiny, being tested, having extra paper work and being under time pressure	results in disengagement
**Inner setting**	Paper 22 Bull et al. (2019) Feasibility of the behaviour change wheel to support clinical teams implementing	Fostering stakeholder engagement through	timely effective 'low tech' feedback, recognition of existing expertise, motivation, communication and education	facilitates ownership and participation leading to participation in initiative

	new models of care			
**Culture**	Paper 22 Bull et al. (2019) Feasibility of the behaviour change wheel to support clinical teams implementing new models of care	Organisations that foster clear roles around new models of care	raise clinician awareness	leading to better change commitment
**Culture**	Paper 22 Bull et al. (2019) Feasibility of the behaviour change wheel to support clinical teams implementing new models of care	Organisations that foster co-development models for implementing change	foster shared ownership and an engaged workforce	leading to sustained change
**Culture**	Paper 22 Bull et al. (2019) Feasibility of the behaviour change wheel to support clinical teams implementing new models of care	Organisations that invest in supporting staff with time	fosters greater motivation	lends to better intra-organisational health partnerships
**Individuals**	Paper 23 Wutzke et al. (2016) Implementation of large scale innovations in complex health care systems: views of managers and frontline personnel	Champions that are trusting and have credibility	generate a tension for change	leading to better implementation commitment
**Process**	Paper 23 Wutzke et al. (2016) Implementation of large scale innovations in complex health care systems: views of managers and frontline personnel	Organisations that provide flexible project management	emphasise outcomes (not timelines)	leading to a sustained implementation
**Culture**	Paper 23 Wutzke et al. (2016) Implementation of large scale innovations in complex health care systems: views of managers and frontline	Organisations that foster conscious raising culture of barriers to an EBP	foster a social environment that fosters solutions	to address implementation barriers

	personnel			
**Culture**	Paper 23 Wutzke et al. (2016) Implementation of large scale innovations in complex health care systems: views of managers and frontline personnel	Organisations that foster a collaborative process	facilitate intervention ownership	inculcate change commitment
**Process**	Paper 23 Wutzke et al. (2016) Implementation of large scale innovations in complex health care systems: views of managers and frontline personnel	Ensuring good support throughout the implementation process requires	a flexible, incremental, outcome focused approach	and leads to better engagement and success
**Culture**	Paper 23 Wutzke et al. (2016) Implementation of large scale innovations in complex health care systems: views of managers and frontline personnel	Stakeholder engagement	fosters cultural change	and influences positive change
**Culture**	Paper 23 Wutzke et al. (2016) Implementation of large scale innovations in complex health care systems: views of managers and frontline personnel	Stakeholder engagement	fosters tension of change	that leads to motivational commitment to implementation
**Intervention characteristics**	Paper 23 Wutzke et al. (2016) Implementation of large scale innovations in complex health care systems: views of managers and frontline personnel	Successful implementation of change may require adaptation by being	flexible, balancing model flexibility and local needs	to maximise collaboration and commitment
**Culture**	Paper 23 Wutzke et al. (2016) Implementation of large scale	Promoting change through stakeholder engagement	by providing support, having champions, creating	leading to staff ownership, advocates and maintainers of

	innovations in complex health care systems: views of managers and frontline personnel		opportunities for staff involvement and acknowledging resistance	the change initiative
**Process**	Paper 24 Williams et al. (2013) Intermediary Programme for infection Control	Intermediaries that feel it is important that staff and patients became used to their ' high visibility' presence on a unit	fosters integrated work relationships	which leads to a more seamless process of quality improvement monitoring, guidance and collective action
**Process**	Paper 24 Williams et al. (2013) Intermediary Programme for infection Control	Intermediaries that adopt monitoring, education, advisory and facilitation roles	are perceived by frontline staff as respectful, skilled, QI role model	resulting in frontline staff approaching them and taking support and advice
**Inner setting**	Paper 24 Williams et al. (2013) Intermediary Programme for infection Control	Intermediaries that provide high level physical presence on clinical units	act as a a reminder for frontline staff of their practice	and promote prompt change to adhere evidence based practice.
**Individuals**	Paper 24 Williams et al. (2013) Intermediary Programme for infection Control	Organisation with a presence of an intermediary	foster an environment of rapport and trust	leading to a collegiate atmosphere, better teamwork and improvements in QI .
**Inner setting**	Paper 24 Williams et al. (2013) Intermediary Programme for infection Control	Intermediaries that conduct practice-based teaching	meet front line staff learning needs in a meaningful way	and results in more knowledgeable and skilled staff.
**Inner setting**	Paper 24 Williams et al. (2013) Intermediary Programme for infection Control	Intermediaries that use facilitative approaches giving feedback (discretely and in context) for performance processes	prompt front line staff to consider their practice	and modify in accordance with evidence
**Outer setting**	Paper 24 Williams et al. (2013) Intermediary Programme for infection Control	Organisation with a presence of an intermediary	gives an enhanced sense of recognition for patients and relatives	leading to public reassurance
**Process**	Riippa et al. (2014) Chronic Care model	Fragmented implementation strategy	fails to provide clear direction in roles and responsibilities for multidisciplinary teams	leading to a loss intervention fidelity.
**Process**	Riippa et al. (2014) Chronic	Implementation planning that	overlooks patient preferences	and leads to less effective

	Care model	fails to engage service users	and values and role in delivery of the intervention	implementation.
**Inner setting**	Riippa et al. (2014) Chronic Care model	Complex interventions require systematic guidelines accompanied with decision support	to enable clinicians gain knowledge and skills in intervention delivery	to commit to change and promote new routine care
**Inner setting**	Riippa et al. (2014) Chronic Care model	When organisations fail to continue to commit leadership engagement, resources (funds and personnel resources) and support	it fosters clinician disillusionment	and hinders the implementation and sustainability of a programme.
**Intervention characteristics**	Paper 26 Noyes et al. (2014) Nurse-led implementation, optimization and evaluation of a complex children's continuing-care policy.	When nurses perceive a new policy intervention as having relative advantage over the existing care	it fosters positivity and change commitment	leading to greater uptake and adoption of an intervention into existing practice.
**Process**	Paper 26 Noyes et al. (2014) Nurse-led implementation, optimization and evaluation of a complex children's continuing-care policy.	Effective implementation leads that engage stakeholders and identify implementation barriers and instigate additional interventions	resolve challenges and meet the need of nurses	making implementation more likely.
**Inner setting**	Paper 26 Noyes et al. (2014) Nurse-led implementation, optimization and evaluation of a complex children's continuing-care policy.	Implementation leads and local facilitators are more successful	when local staff perceive their feedback is meaningful and making a difference in the implementation planning	leading to change motivation and commitment among staff.
**Inner setting**	Paper 26 Noyes et al. (2014) Nurse-led implementation, optimization and evaluation of a complex children's continuing-care policy.	Insufficient existing structures require resourcing for service reorganisation and investment	to ensure organisational & staff capacity and capability	to implement an intervention.
**Process**	Paper 26 Noyes et al. (2014)	Organisations that undertake	and respond to their opinions	are more likely realistic 'fit'

	Nurse-led implementation, optimization and evaluation of a complex children's continuing-care policy.	'real-time' consultation with stakeholders about intervention compatibility	by adapting and revising an intervention while maintaining fidelity	with what nurses considered would work with within their service.
**Process**	Paper 26 Noyes et al. (2014) Nurse-led implementation, optimization and evaluation of a complex children's continuing-care policy.	Implementation planning that lacks clarity of processes can	can foster a hostile contextual condition where stakeholders fail to share the same expectations	leading to challenges with implementation
**Individuals**	Paper 27 Craig (2016) The barriers and enablers for a triage, treatment, and transfer clinical intervention to manage acute stroke patients in the emergency department.	Staff lack of education and awareness of evidence-based treatment (stroke)	were provided with training and education and also included CPD	that resulted in improved stroke care by increased uptake of evidence-based treatment
**Inner setting**	Paper 27 Craig (2016) The barriers and enablers for a triage, treatment, and transfer clinical intervention to manage acute stroke patients in the emergency department.	Staff lack of accessibility to pathways and protocols	was improved by access to policies and protocols in the settings	Increased uptake and improve stroke management
**Cultural**	Paper 27 Craig (2016) The barriers and enablers for a triage, treatment, and transfer clinical intervention to manage acute stroke patients in the emergency department.	Lack of recognition of Social/professional role Limited social identity, i.e., insufficient recognition by peers and decision makers	Good leadership -champion identified , Verbal persuasion about capability and meetings	Increased uptake of evidence-based management (stroke)
**Individuals**	Paper 27 Craig (2016) The barriers and enablers for a triage, treatment, and transfer clinical intervention to manage acute stroke patients in the emergency department.	Beliefs about consequences of intervention	stemmed from old- fashioned views on stroke, belief re positive outcomes not present	Reluctance to administer intervention

**Inner setting**	Paper 27 Craig (2016) The barriers and enablers for a triage, treatment, and transfer clinical intervention to manage acute stroke patients in the emergency department.	Working with a busy environment and stressful working conditions	MDT cooperation and support and sharing experience	Increased uptake - Communication between departments was believed to play a pivotal role in the success of intervention
**Inner setting**	Paper 29 McInnes (2020) Implementing continuity of midwife care	Poor communication	obviated by leadership and effective support from the management	Trusting relationships across all organisational levels works triggered a commitment to provide high quality care; if not - sustained or constrained implementation. Midwives felt responsibility -change approaches. Could increase anxiety if not good experience
**Process**	Paper 29 McInnes (2020) Implementing continuity of midwife care	Organisation has current fragmented care process	Implemented visible and supportive leadership	midwives feel safe, valued and informed, supported learning and able to ask questions
**Process**	Paper 29 McInnes (2020) Implementing continuity of midwife care	Continuity v current fragmented care processed	Implemented visible and supportive leadership put in place during implementation	changed midwives perspective of how they provided care and of women's care need.
**Individuals**	Paper 29 McInnes (2020) Implementing continuity of midwife care	Inconsistencies in beliefs at all levels	Build professional relationships (frequent care contacts with the same midwife during a woman's care journey) and collaborative working.	Felt safe and able to engage
**Individuals**	Paper 29 McInnes (2020) Implementing continuity of midwife care	Midwives need to be in control of their own workload to be able to practise flexibly and autonomously	Prioritising space & time for team meetings-a shared vision and commitment across the workforce	Role satisfaction
**Cultural**	Paper 30 Buswell (2017) Managing Faecal Incontinence in Care Home Residents Living	Staff had shared understanding of the problem	Access to education, training and feedback	Knowledge and understanding increased leading to a change in practice

	With Dementia			
**Process**	Paper 30 Buswell (2017) Managing Faecal Incontinence in Care Home Residents Living With Dementia	Lack of time for training and mentoring	Training programme offered to staff and family	Staff and family working together leads to better patient outcome
**Inner setting**	Paper 30 Buswell (2017) Managing Faecal Incontinence in Care Home Residents Living With Dementia	Clinical assessment fragmented/missing	Became integral to the work patterns	reduction in episodes of FI, reduction in resident distress, family caregiver satisfaction with care, staff confidence, costs
**Culture**	Paper 30 Buswell (2017) Managing Faecal Incontinence in Care Home Residents Living With Dementia	Task orientated	Person-centered care training and structured programs	lead to staff engagement and motivation to change practice. Changed patient environment - toilets, tools, visual aids, prompts, etc
**Inner setting**	Paper 30 Buswell (2017) Managing Faecal Incontinence in Care Home Residents Living With Dementia	Organisational workload environment	time for training allowed, change in documentation	Improved knowledge and confidence
**Inner setting**	Paper 30 Buswell (2017) Managing Faecal Incontinence in Care Home Residents Living With Dementia	Organisational structure	Managerial permission to move to patient centered care, reflective approach	less distress for residents, staff satisfaction, family care-giver satisfaction with care
**Inner setting**	Paper 30 Buswell (2017) Managing Faecal Incontinence in Care Home Residents Living With Dementia	Engagement and encouragement of leadership team	Promote staff discussion. Staff to believe that change is possible	Staff are enabled and accept/engage with intervention
**Intervention characteristics**	Paper 31 Sopcak et al. (2016) Exploring barriers and facilitators of a novel way to improve chronic disease prevention and screening in primary care	Complexity (paperwork and time) and cost of the intervention	Perceived as overwhelming and time consuming by practitioners	Barrier to implementation for practitioners; patients appreciated time and treated this as a way of being treated comprehensively i.e., holistic and multi-factorial way

**Inner setting**	Paper 31 Sopcak et al. (2016) Exploring barriers and facilitators of a novel way to improve chronic disease prevention and screening in primary care	Unclear responsibilities/boundaries creates tension and discomfort	Establishing good working relationships and trust, working as a team	Facilitates better teamwork and implementation
**Intervention characteristics**	Paper 31 Sopcak et al. (2016) Exploring barriers and facilitators of a novel way to improve chronic disease prevention and screening in primary care	Intervention program fits with professional mandate/hot topic in policy (credibility of the programme in quality and strength of evidence)	Staff value and see it as a good fit with clinical role	Facilitates program engagement/implementation
**Process**	Paper 31 Sopcak et al. (2016) Exploring barriers and facilitators of a novel way to improve chronic disease prevention and screening in primary care	Ability to support and motivate patients	Places patient in driver seat to take ownership of roles	Patients feel supported and motivated
**Process**	Paper 31 Sopcak et al. (2016) Exploring barriers and facilitators of a novel way to improve chronic disease prevention and screening in primary care	Lack of local champion	Availability of local champion	Facilitates program implementation
**Process**	Paper 31 Sopcak et al. (2016) Exploring barriers and facilitators of a novel way to improve chronic disease prevention and screening in primary care	Early engagement of administrators and managers / collaborative and teamwork connections (planning and engaging process)	Increase stakeholder engagement and optimise communication; with ability to better adapt program to need of participants	Improvement in uptake /desire to join project; clarification of role, scope, expectations
**Process**	Paper 31 Sopcak et al. (2016) Exploring barriers and facilitators of a novel way to	Competing health care demands and limited resources especially lack of staff	Made if difficult to allocate time or resources	Hindered engagement with the programme/implementation

	improve chronic disease prevention and screening in primary care			
**Culture**	Paper 32 Abhyankar et al. (2013) Normal Birth Programme (note links with paper 1).	Medicalised culture, unequal power and authority between midwives and obstetricians	Resistance to change, feel top down, perceived erosion of power	Low engagement from stakeholders, difficulties facilitating, tough strategies
**Culture**	Paper 32 Abhyankar et al. (2013) Normal Birth Programme (note links with paper 1).	Pro-normality culture, midwives recognised as equals	Less resistance to change, stakeholders involved though distant	High engagement from stakeholders, smoother facilitation
**Intervention characteristics**	Paper 32 Abhyankar et al. (2013) Normal Birth Programme (note links with paper 1).	Subtle implementation – pathways integrated and adapted to local guidelines	No obvious requirement for change	Greater use of pathways and adherence to KCND principles
**Individuals**	Paper 33 Eldh et al. (2014) Facilitators and barriers to applying a national quality registry (NQR) for quality improvement in stroke care	Local stakeholders knowledge of and interest in stroke and QI	Facilitated learning about the registry and stroke care. Can apply the NQR data to effectively initiate, carry out, and evaluate quality improvement	Promotes implementation of local quality improvement initiatives
**Process**	Paper 33 Eldh et al. (2014) Facilitators and barriers to applying a national quality registry (NQR) for quality improvement in stroke care	Collaboration (internal) with and supported by managers and co-workers / engagement of upper management	Stimulated staff and engaged them by managers showing interest in output data that illustrated evidence of improvements and identified issues of relevance to the everyday stroke care provided in the unit.	Promotes implementation of local quality improvement initiatives
**Intervention characteristics**	Paper 33 Eldh et al. (2014) Facilitators and barriers to applying a national quality registry (NQR) for quality	A common stroke care process, with NQR variables linking to national guidelines	Staff trusted guidelines that were in line with agreed evidence in national guidelines	Trustworthiness of NQR variables indicated to stakeholders that output data were reliable and could be used

	improvement in stroke care			to inform local system; helped convince staff to record patient data properly
**Outer setting**	Paper 33 Eldh et al. (2014) Facilitators and barriers to applying a national quality registry (NQR) for quality improvement in stroke care	External collaborations with networks regionally and nationally	Meeting others engaged in stroke and Ribs-Stroke in regional networks was inspiring; annual NQR meetings; opportunities for sharing ideas	Facilitates local QI; output data for each hospital's stroke unit became source for local mapping of adherence to evidence/benchmark
**Intervention characteristics**	Paper 33 Eldh et al. (2014) Facilitators and barriers to applying a national quality registry (NQR) for quality improvement in stroke care	NQR considered relevant and credible, a source of evidence, and must be applied in the local context.	Having resources to manage local data; stakeholders know how to initiate, perform, and evaluate quality improvement, and have the resources to do so, including time	Informs local management systems
**Inner setting**	Paper 33 Eldh et al. (2014) Facilitators and barriers to applying a national quality registry (NQR) for quality improvement in stroke care	Credible and competent staff working with Risk-Stroke at the stroke unit	Time to meet, discuss	Embrace and engage with NQR data; If questioned or unsupported, experienced a decrease of engagement with NQR; if not in stroke unit - less implementation as perceived more work involved
**Resources**	Paper 34 Gordon et al. (2018) Optimal healthcare delivery to care homes in the UK	Investment at institutional level i.e. funding; presence of funding and institutional endorsement (resources); seen as legitimate use of time/skills	Enabled staff (visiting healthcare practitioners) being allocated time to work in care homes; triggered a commitment and willingness to work proactively with care home staff	Positive outcomes with services engaging and maintenance of residents in care homes
**Inner setting**	Paper 34 Gordon et al. (2018) Optimal healthcare delivery to care homes in the UK	Relational working builds over time	Shared learning and persistence, time and space allocated, mutual professional development; sense of common	Becomes embedded and recognised as normal practice; practitioners confident can provide or access services

			endeavour and generated willingness to work together	
**Process**	Paper 35 McConnell (2013) Key Factors Affecting the Successful Implementation and Sustainability of the Liverpool Care Pathway for the Dying Patient	Resources e.g. lack of facilitator, time	**A dedicated facilitator**: provided training for staff in how to communicate recognition that the patient is dying with both patients and relatives; providing feedback on positive outcomes of pathway usage -using literature; provided reassurance to staff over the withdrawal of unnecessary routine practice such as measuring blood pressure.	Success of LCP: motivated staff; increased confidence in staff to implement the pathway. Facilitation appears to work by making staff aware of the goals of the LCP, providing reassurance in terms of their skills and decision-making in early implementation. This leads to a self-perception of competency and capacity, which increases the likelihood that new approaches are embedded, unnecessary practices are stopped, and communication takes place with relatives and patients.
**Individuals**	Paper 35 McConnell (2013) Key Factors Affecting the Successful Implementation and Sustainability of the Liverpool Care Pathway for the Dying Patient	Beliefs of staff in relation to end of life care: curative versus palliative care	**Education, training and support**	Motivated staff to improve care, sustained implementation; increased competence and capacity; new competencies and beliefs among staff on why and how to use the pathway. Education created more confidence and openness in staff to discuss death and dying. This in turn improved communication and collaboration amongst the MDT and with patients and their relatives.

**Inner setting**	Paper 35 McConnell (2013) Key Factors Affecting the Successful Implementation and Sustainability of the Liverpool Care Pathway for the Dying Patient	Is present situation acceptable?	**Audit:** Pre-implementation audit: Identified deficiencies in service. Post-implementation audit. Feedback. National audit.	Audit: changed doctors' and nurses' perceptions of the pathway and encouraged uptake and sustainability. National audit helped secure funding for education and training
**Inner setting**	Paper 35 McConnell (2013) Key Factors Affecting the Successful Implementation and Sustainability of the Liverpool Care Pathway for the Dying Patient	Support of senior managers	**Resources given:** Time for training, for audit and feedback, money and facilitation. Training mandatory for all MDT	Everyone had common understanding, better implementation
**Outer setting**	Paper 36 Jeff et al. (2017) Identifying Effective Nurse-Led Care Transition Interventions for Older Adults With Complex Needs	Patient resources may be limited	Determined preferred level of involvement, readiness and capabilities of patients, their family members, and/or caregivers. Engaged them in co-design of care transition interventions; Educated them. Made aware of follow-up	Will empower patients etc and result in quality care transitions experiences and outcomes
**Inner setting**	Paper 36 Jeff et al. (2017) Identifying Effective Nurse-Led Care Transition Interventions for Older Adults With Complex Needs	Staff resources may be limited: ability to lead, time availability, insufficient knowledge	Educate nurses re their new role. Optimize nurses' role by facilitating MDT comprehensive holistic assessment. Create more specialized care transition nursing roles and education to MSc etc	Will empower staff
**Networks and communications**	Paper 36 Jeff et al. (2017) Identifying Effective Nurse-Led Care Transition Interventions for	Systems/organisational barriers: healthcare systems operating in silos.	Use of standardized documentation tools and comprehensive communication strategies. Build in feedback	Will result in quality care transitions experiences and outcomes for the complex older person population. Will

	Older Adults With Complex Needs		systems that ensure receipt of reports.	maintain<br>the primary health record;
**Inner setting**	Paper 36 Jeff et al. (2017) Identifying Effective Nurse-Led Care<br>Transition Interventions for Older Adults With Complex Needs	Organizational commitment for changes in practice.	Put in strong leadership, strategic alignment and accountability structures	Will lead to intervention success
**Outer setting**	Paper 37 Hooft et al. (2017) what do nurse-led self-management interventions achieve for outpatients with a chronic condition?	Perceived need to support patients	Group meetings. Personalised information. Promoted use of reflective diaries	Patient coping skills increased. Little success with diary
**Outer setting**	Paper 37 Hooft et al. (2017) what do nurse-led self-management interventions achieve for outpatients with a chronic condition?	Perceived need to support newly diagnosed patients separately	Education focused on managing day-to-day problems and "how to" skills. Use of individual, group and family sessions	Increase in self-efficacy and patients felt more supported in daily life. Additional instruction of family members not effective.
**Culture**	Paper 37 Hooft et al. (2017) what do nurse-led self-management interventions achieve for outpatients with a chronic condition?	Culturally and linguistically diverse groups	Use interpreters	Less successful in increasing self care
**Intervention characteristics**	Paper 37 Hooft et al. (2017) what do nurse-led self-management interventions achieve for outpatients with a chronic condition?	Belief that Intervention mechanisms must be based on theory	Theory of each intervention outlined	Provides guidance for staff
