# Supplementary material for: A Rapid Realist Review of Quality Care Process Metrics Implementation in Nursing and Midwifery Practice
Source: Int J Environ Res Public Health. 2021 Nov 13;18(22):11932. doi: 10.3390/ijerph182211932 (PMC8621300; doi:10.3390/ijerph182211932)
Supplement: Supplementary file 1 [file ijerph-18-11932-s001.zip › 3.Supplementary Table S3 Included Documents.pdf]

Supplementary File S3

Table S3. Included Documents with Secondary Research Studies highlighted

Document Number	Authors	Title	Year	Country of Origin	Methodology	Reference to				
						Nursing	Midwifery	Nursing & Midwifery	Policy	Specific Quality Measurement Initiative/s
1	Cheyne, H., Abhyanker, P. and McCourt, C.	Empowering change: Realist evaluation of a Scottish Government programme to support normal birth.	2013	United Kingdom	Realist Evaluation		X		X	
2	Francis-Coad, J., Etherton-Beer, C., Bulsara, C., Blackburn, N., Chivers, P. and Hill, A.	Evaluating the impact of a falls prevention community of practice in a residential aged care setting: a realist approach.	2018	Australia	Mixed Methods	X			X	X
3	Stalpers, D., De Vos, M.L., Van Der Linden, D., Kaljouw, M. J. and Schuurmans, M. J.	Barriers and carriers: a multicenter survey of nurses' barriers and facilitators to monitoring of nurse-sensitive outcomes in intensive care units	2017	Netherlands	Multi-Centre Survey Questionnaire			X		X
4	Sims, S., Leamy, M., Davies, N., Schnitzler, K., Levenson, R., Mayer, F., Grant, R., Brearley, S., Gourlay, S., Ross, F. and Harris, R.	Realist synthesis of intentional rounding in hospital wards: exploring the evidence of what works, for whom, in what circumstances and why.	2018	United Kingdom	Realist Synthesis					
5	Cross, B. and Cheyne, H.	Strength-based approaches: a realist evaluation of implementation in maternity services in Scotland.	2018	United Kingdom	Realist Evaluation			X		
6	Williams, L., Rycroft-Malone, J. and Burton, C. R.	Implementing best practice in infection prevention and control. A realist evaluation of the role of intermediaries.	2016	United States of America	Realist Evaluation	X			X	
7	Avra, S., Janet D., Ian C., Jacques L. and Yona L.	Original qualitative research barriers and facilitators to improving health care for adults with intellectual and developmental disabilities: what do staff tell us?	2018	Canada	Qualitative Study	X			X	
8	Bunn, F., Goodman, C., Russell, B., Wilson, P., Manthorpe, J., Rait, G., Hodkinson, I. and Durand,	Supporting shared decision making for older people with multiple health and social care needs: a realist synthesis.	2018	United Kingdom	Realist Synthesis	X			X	X

Document Number	Authors	Title	Year	Country of Origin	Methodology	Reference to				
						Nursing	Midwifery	Nursing & Midwifery	Policy	Specific Quality Measurement Initiative/s
	M.									
9	Gee, M., Bhanbhro, S., Cook, S. and Killaspy, H.	Rapid realist review of the evidence: achieving lasting change when mental health rehabilitation staff undertake recovery-oriented training.	2017	United Kingdom	Rapid Realist Review	X			X	
10	Brown, S., Lhussier, M., Dalkin, S. M. and Eaton, S.	Care Planning: What Works, for Whom, and in What Circumstances? A Rapid Realist Review.	2018	United Kingdom	Rapid Realist Review	X			X	X
11	Li, S. A., Jeffs, L., Barwick, M. and Stevens, B.	Organizational contextual features that influence the implementation of evidence-based practices across healthcare settings: a systematic integrative review.	2018	Canada	Systematic Integrative Review			X	X	X
12	Bryce, C., Fleming, J. and Reeve, J.	Implementing change in primary care practice: lessons from a mixed-methods evaluation of a frailty initiative.	2018	United Kingdom	Mixed Methods	X			X	X
13	Pearson, M., Brand, S. L., Quinn, C., Shaw, J., Maguire, M., Michie, S., Briscoe, S., Lennox, C., Stirzaker, A., Kirkpatrick, T. and Byng, R.	Using realist review to inform intervention development: methodological illustration and conceptual platform for collaborative care in offender mental health.	2015	United Kingdom	Realist Review				X	
14	Yamada, J., Potestio, M. L.., Cave, A. J., Sharpe, H., Johnson, D. W., Patey, A. M., Presseau, J. and Grimshaw, J. M.	Using the theoretical domains framework to identify barriers and enablers to paediatric asthma management in primary care settings.	2018	Canada	Qualitative Study	X				X
15	Baatiema, L., Otim, M. E., Mnatzaganian, G., de-Graft Aikins, A., Coombes, J. and Somerset, S.	Health professionals' views on the barriers and enablers to evidence-based practice for acute stroke care: a systematic review.	2017	Australia	Systematic Review	X			X	
16	McCluskey, A., Vratsistas-Curto, A. and Schurr, K.	Barriers and enablers to implementing multiple stroke guideline recommendations: a qualitative study.	2013	Australia	Qualitative Study	X			X	
17	Foster, A., Croot, L., Brazier, L., Harris, J. and O'Cathain, A.	The facilitators and barriers to implementing patient reported outcome measure sin organisations	2018	United Kingdom	Systematic Review of Reviews				X	

Document Number	Authors	Title	Year	Country of Origin	Methodology	Reference to				
						Nursing	Midwifery	Nursing & Midwifery	Policy	Specific Quality Measurement Initiative/s
		delivering health related services: a systematic review of reviews.								
18	McHugh, M., Brown, T., Liss, D. T., Walunas, T. L. and Persell, S. D.	Practice Facilitators' and Leaders' Perspectives on a Facilitated Quality Improvement Program.	2018	United States of America	Qualitative Study					
19	Hanson, H. M., Warkentin, L., Wilson, R., Sandhu, N., Slaughter, S. E. and Khadaroo, R. G.	Facilitators and barriers of change toward an elder-friendly surgical environment: perspectives of clinician stakeholder groups.	2017	Canada	Mixed Methods	X			X	
20	Bee, P., Price, O., Baker, J. and Lovell, K.	Systematic synthesis of barriers and facilitators to service user-led care planning.	2015	United Kingdom	Systematic Synthesis	X			X	
21	Smith, T., McNeil, K., Mitchell, R., Boyle, B. and Ries, N.	A study of macro-, meso- and micro-barriers and enablers affecting extended scopes of practice: the case of rural nurse practitioners in Australia.	2019	Australia	Qualitative Study			X	X	
22	Bull, E. R., Hart, J. K., Swift, J., Baxter, K., McLauchlan, N., Joseph, S. and Byrne-Davis, L. M. T.	An organisational participatory research study of the feasibility of the behaviour change wheel to support clinical teams implementing new models of care.	2019	United Kingdom	Participatory Research Study			X	X	
23	Wutzke, S., Benton, M. and Verma, R.	Towards the implementation of large scale innovations in complex health care systems: views of managers and frontline personnel.	2016	Australia	Qualitative Study					
24	Williams, L., Burton, C. and Rycroft-Malone, J.	What works: a realist evaluation case study of intermediaries in infection control practice.	2013	United States of America	Realist Evaluation	X			X	
25	Riippa, I., Kahilakoski, O., Linna, M. and Hietala, M.	Can complex health interventions be evaluated using routine clinical and administrative data? - a realist evaluation approach.	2014	Finland	Realist Evaluation	X				
26	Noyes, J., Lewis, M., Bennett, V., Widdas, D. and Brombley, K.	Realistic nurse-led policy implementation, optimization and evaluation: novel methodological exemplar.	2014	United Kingdom	Implementation & Evaluation design with Realist Principles			X	X	

Document Number	Authors	Title	Year	Country of Origin	Methodology	Reference to				
						Nursing	Midwifery	Nursing & Midwifery	Policy	Specific Quality Measurement Initiative/s
27	Craig, L. E., McInnes, E., Taylor, N., Grimley, R., Cadilhac, D. A., Considine, J. and Middleton, S.	Identifying the barriers and enablers for a triage, treatment, and transfer clinical intervention to manage acute stroke patients in the emergency department: a systematic review using the theoretical domains framework (TDF).	2016	Australia	Systematic Review	X			X	
28	Clemson, L., Laver, K., Jeon, Y. H., Comans, T. A., Scanlan, J., Rahja, M., Culph, J., Low, L. F., Day, S., Cations, M., Crotty, M., Kurrle, S., Piersol, C. and Gitlin, L. N.	Implementation of an evidence-based intervention to improve the wellbeing of people with dementia and their carers: study protocol for 'Care of People with dementia in their Environments (COPE)' in the Australian context.	2018	Australia	Mixed Methods	X			X	
29	McInnes, R. J., Aitken-Arbuckle, A., Lake, S., Hollins, M. C. and MacArthur, J.	Implementing continuity of midwife carer - just a friendly face? A realist evaluation.	2020	Australia	Realist Evaluation		X			X
30	Buswell, M., Goodman, C., Roe, B., Russell, B., Norton, C., Harwood, R., Fader, M., Harari, D., Drennan, V. M., Rycroft Malone, J., Madden, M. and Bunn, F.	What Works to Improve and Manage Fecal Incontinence in Care Home Residents Living With Dementia? A Realist Synthesis of the Evidence.	2017	United States of America	Realist Synthesis	X			X	X
31	Sopcak, N., Aguilar, C., O'Brien, M. A., Nykiforuk, C., Aubrey-Bassler, K., Cullen, R., Grunfeld, E. and Manca, D. P.	Implementation of the BETTER 2 program: a qualitative study exploring barriers and facilitators of a novel way to improve chronic disease prevention and screening in primary care.	2016	Canada	Qualitative Study	X			X	
32	Abhyankar, P., Cbeyne, H., Maxwell, M., Harris, A. F. and McCourt, C.	A realist evaluation of a normal birth programme.	2013	United Kingdom	Realist Evaluation			X	X	
33	Eldh, A. C., Fredriksson, M., Halford, C., Wallin, L., Dahlström, T., Vengberg, S. and Winblad, U.	Facilitators and barriers to applying a national quality registry for quality improvement in stroke care.	2014	Sweden	Mixed Methods	X				X
34	Gordon, A. L., Goodman, C.	Optimal healthcare delivery to care	2018	United	Realist	X			X	

Document Number	Authors	Title	Year	Country of Origin	Methodology	Reference to				
						Nursing	Midwifery	Nursing & Midwifery	Policy	Specific Quality Measurement Initiative/s
	L., Davies, S., Dening, T., Gage, H., Meyer, J., Schneider, J., Bell, B., Jordan, J., Martin, F. C., Illiffe, S., Bowman, C., Gladman, J. R. F., Victor, C., Mayrhofer, A., Handley, M. and Zubair, M.	homes in the UK: a realist evaluation of what supports effective working to improve healthcare outcomes.		Kingdom	Evaluation					
35	McConnell, T., O'Halloran, P., Porter, S. and Donnelly, M.	Systematic Realist Review of Key Factors Affecting the Successful Implementation and Sustainability of the Liverpool Care Pathway for the Dying Patient.	2013	United Kingdom	Systematic Realist Review	X			X	X
36	Jeffs, L., Kuluski, K., Law, M., Saragosa, M., Espin, S., Ferris, E., Merkley, J., Dusek, B., Kastner, M. and Bell, C. M.	Identifying Effective Nurse-Led Care Transition Interventions for Older Adults With Complex Needs Using a Structured Expert Panel.	2017	Canada	Modified Delphi Consensus Technique based on the RAND method	X			X	X
37	Hooft, S. M., Been - Dahmen, J. M. J., Ista, E., Staa, A. and Boeije, H. R.	A realist review: what do nurse-led self-management interventions achieve for outpatients with a chronic condition?	2017	Netherlands	Realist Review	X			X	X
