# Supplementary material for: A Rapid Realist Review of Quality Care Process Metrics Implementation in Nursing and Midwifery Practice
Source: Int J Environ Res Public Health. 2021 Nov 13;18(22):11932. doi: 10.3390/ijerph182211932 (PMC8621300; doi:10.3390/ijerph182211932)
Supplement: Supplementary file 1 [file ijerph-18-11932-s001.zip › 4.Supplementary Table S4 CMOc and PT Refinement.pdf]

Supplemental File S4

Table S4. CMOc and Programme Theory Refinement

**CMOc Feeding into Programme Theory 1**	**Source**
Context: Deployment - staged or system wide affected<br>*Reaction:* how/when staff engaged with the programme<br>*Outcome:* enabling staff to use the programme to facilitate improvement in patient outcomes	(Cheyne, Abhyankar and McCourt, 2013)
Context: Introduction of the policy tools to service users evidence-based midwifery practices<br>*Resource*: was supported by gradual rather than abrupt intervention, by use of standarised records, care pathways and risk assessments<br>*Outcome*: that developed Midwifes' motivation to change and promoted equality in care provision leading to some patients' (women's) active engagement; however the degree of engagement was influenced by socio economic factors of patients.	(Cheyne et al., 2013)
Context: Complexity (paperwork and time) and cost of the intervention<br>*Reaction*: Perceived as overwhelming and time consuming by practitioners<br>*Outcome*: Barrier to implementation for practitioners; patients appreciated time and treated this as a way of being treated comprehensively i.e., holistic and multi-factorial way	(Cross and Cheyne, 2018)
Context: Intervention program fits with professional mandate/hot topic in policy (credibility of the programme in quality and strength of evidence)<br>*Reaction*: Staff value this and see it as a good fit with clinical role<br>*Outcome*: Facilitates program engagement/implementation	(Cross and Cheyne, 2018)
Context: Subtle implementation – pathways integrated and adapted to local guidelines<br>*Reaction*: No obvious requirement for change<br>*Outcome*: Greater use of pathways and adherence to KCND principles	(Cross and Cheyne, 2018)
Context: A common stroke care process, with NQR variables linking to national guidelines<br>*Resource & Reaction*: Staff trusted guidelines that were in line with agreed evidence in national guidelines<br>*Outcome*: Trustworthiness of NQR variables indicated to stakeholders that output data were reliable and could be used to inform local system; helped convince staff to record patient data properly	(Cross and Cheyne, 2018)
Context: NQR considered relevant and credible, a source of evidence, and must be applied in the local context.<br>*Resource*: Having resources to manage local data; stakeholders know how to initiate, perform, and evaluate quality improvement, and	(Cross and Cheyne, 2018)

have the resources to do so, including time<br>*Outcome*: Informs local management systems	
Context: Belief that Intervention mechanisms must be based on theory<br>*Resource*: Theory of each intervention outlined<br>*Outcome*: Provides guidance for staff	(Cross and Cheyne, 2018)

**CMOc Feeding into Programme Theory 2**	**Source**
Context: Support for programme at national and local level<br>*Resource & Reaction*: facilitated by the presence of resources and local champions<br>*Outcome*: enabled midwifes to focus on implementation	(Cheyne et al., 2013)
Context: Busy work environment/workload was obviated by<br>*Resource*: incorporating the Nurse sensitive outcomes (NSO) monitoring into nurses' regular duties and responsibilities<br>*Outcome*: so that NSOs were not seen as additional work and burden	(Cheyne et al., 2013)
Context: Different stages of training and implementation across health boards<br>*Reaction*: led to correspondingly different approaches to patient assessment by Midwifes<br>*Outcome*: that resulted in the midwives experiential learning being a key factor in motivating longer-term implementation.	(Cheyne et al., 2013)
Context: Caseload variance from health board to health board<br>*Reaction*: required joint working across the health boards to develop materials for programme implementation<br>*Outcome*: which meant variation in implementation lead to consequential difference in Midwifes' experience of EBP	(Cheyne et al., 2013)
Context: An International evidence-based approach<br>*Reaction*: required joint working across the health boards to develop materials for programme implementation<br>*Outcome*: which meant variation in implementation lead to consequential difference in Midwifes' experience of EBP	(Cheyne et al., 2013)
Context: Where the QI strategy was supported by the National Implementation Research Network<br>*Reaction*: staff engaged with provided resources (e.g. implementation logs/champions/electronic reminders and prompts)<br>*Outcome*: resulting in increased chance of intervention success	(Francis-Coad *et al.*, 2018)
Context: External collaborations with networks regionally and nationally<br>*Resource*: Meeting others engaged in stroke and Risks-Stroke in regional networks was inspiring; annual NQR meetings; opportunities for sharing ideas<br>*Outcome*: Facilitates local QI; output data for each hospital's stroke unit became source for local mapping of adherence to evidence/benchmark	(Cross and Cheyne, 2018)

**CMOc Feeding into Programme Theory 3**	**Source**
Context: Programme was integrated with existing local study site protocols<br>*Resource:* and negotiated for local use with the multidisciplinary team<br>*Outcome:* leading to standardised practice, midwifery led care and adherence to principles	(Cheyne et al., 2013)
Context: Good midwifery practice existed within the study sites<br>*Resource & Reaction:* so the intervention was adapted to compliment local midwifery practice<br>*Outcome:* resulting in a perceived increase in efforts towards normality and reduction in intervention rates	(Cheyne et al., 2013)
Context: An existing practice culture<br>*Resource & Reaction:* resulted in pathways being used heuristically<br>*Outcome:* that resulted in obstetricians being supportive and midwifes perceived as equals	(Cheyne et al., 2013)
Context: Organisational culture of management support<br>*Reaction:* prioritised staff participation in the falls prevention programme<br>*Outcome:* enabling the implementation of evidenced based falls prevention strategies	(Cheyne et al., 2013)
Context: Existing communication structures<br>*Resource:* used a feedback loop to report on the actions of programme implementation<br>*Outcome:* which triggered proactive behaviour and focused attention on the falls prevention programme	(Cheyne et al., 2013)
Context: Existing workloads<br>*Reaction:* hampered staff engagement with the falls prevention programme<br>*Outcome:* meaning staff did not participate in the programme	(Cheyne et al., 2013)
Context: Use of a community of practice<br>*Resource & Reaction:* brought together likeminded people<br>*Outcome:* enabling the implementation of evidenced based falls prevention strategies	(Cheyne et al., 2013)
Context: Staff receptivity to falls prevention programme<br>*Resource:* was facilitated by available education and training,<br>feedback loop, knowledge sharing and social learning opportunities<br>*Outcome:* enabling the implementation of evidenced based falls prevention strategies	(Cheyne et al., 2013)
Context: Busy work environment/workload was obviated by<br>*Resource:* incorporating the Nurse sensitive outcomes (NSO) monitoring into nurses' regular duties and responsibilities<br>*Outcome:* so that NSOs were not seen as additional work and burden	(Cheyne et al., 2013)
Context: Practice environment supported by<br>*Resource:* practitioner autonomy and feedback loops<br>*Outcome:* leading to satisfaction with work environment	(Cheyne et al., 2013)

Context: Busy work environment/workload was obviated by<br>*Reaction:* staff who engaged with programme as they saw the benefits for patient care and family communication<br>*Outcome:* which led to increased staff vigilance and awareness of psychological and physical patient needs	(Cheyne et al., 2013)
Context: Other competing initiatives were obviated by<br>*Reaction:* staff who engaged with programme as they saw the benefits for patient care and family communication<br>*Outcome:* enabling workload organisation for nursing staff and their ability to<br>anticipate person requirements	(Cheyne et al., 2013)
Context: Existing practice culture (embedded) facilitated<br>*Resource:* provision of time for intentional rounding<br>*Outcome:* until it became 'business as usual'/standard practice	(Cheyne et al., 2013)
Context: Passive leadership endorsement<br>*Resource & Reaction:* discouraged staff engagement<br>*Outcome:* leading to variability in adoption of new practices	(Francis-Coad et al., 2018)
Context: Low morale and high staff turnover<br>*Resource & Reaction:* discouraged staff engagement<br>*Outcome:* leading to variability in adoption of new practices	(Francis-Coad et al., 2018)
Context: Variability in available resources<br>*Reaction:* led to variability in staff engagement with the intervention<br>*Outcome:* leading to corresponding variation in degree of engagement with intervention	(Francis-Coad et al., 2018)
Context: Competing initiatives<br>*Reaction:* meant staff had limited time to engage with the intervention<br>*Outcome:* leading to lack of or passive engagement with intervention	(Francis-Coad et al., 2018)
Context: Job uncertainty and fear<br>*Reaction:* hindered staff engagement, enthusiasm and change 'receptiveness'<br>*Outcome:* resulting in a lack of sustainable change of practice	(Francis-Coad et al., 2018)
Context: Modifying organisational structures to support change<br>*Resource:* helped staff members feel supported by their peers and managers in the change programme<br>*Outcome:* resulting in a sustainable change of practice	(Francis-Coad et al., 2018)
Context: Organisational openness to trialling new ideas and learning<br>*Resource: & Reaction:* meant staff engaged with new evidence based practice (EBP)<br>*Outcome:* increasing the success of new EBP implementation	(Francis-Coad et al., 2018)
Context: Transformational leadership supportive of staff<br>*Resource & Reaction:* facilitated staff self-perception as part of the implementation team	(Francis-Coad et al., 2018)

Outcome: leading to a learning culture and sustainable change	
Context: Leadership that was unsupportive of change or willing to hold staff accountable for change<br>*Reaction:* meant staff felt unmotivated to change<br>*Outcome:* leading to failure of new EBP implementation	(Francis-Coad et al., 2018)
Context: Variation in availability of financial resources across different departments and sites<br>*Resource &Reaction:* meant variation in staff access to EBP supports<br>*Outcome:* resulting in staff struggling to provide adequate service	(Francis-Coad et al., 2018)
Context: Staffing and workload demands<br>*Reaction:* meant staff were less likely to engage with change<br>*Outcome:* leading to failure of new EBP implementation	(Francis-Coad et al., 2018)
Context: Time and competing demands<br>*Reaction:* meant staff were unable to engage with or failed to prioritise new EBP<br>*Outcome:* leading to failure of new EBP implementation	(Francis-Coad et al., 2018)
Context: Poor communication to staff<br>*Reaction:* meant felt unclear about where responsibility ended, both in terms of accountability for clinical decisions, and in defining the limits of care<br>*Outcome:* resulting in ongoing uncertainty that limited further implementation	(Francis-Coad et al., 2018)
Context: Development of a service model to suit local needs<br>*Resource:* with no local supports in place for staff to engage with<br>*Outcome:* leading to significant levels of sickness/ absence in some teams (but correlation rather than causation established).	(Francis-Coad et al., 2018)
Context: There was an infrastructure to support practitioners work<br>*Resource:* that gave practitioners the opportunity to develop their skills and have their practice supported<br>*Outcome:* resulting in monitoring of individuals in need of care, and the provision of feedback and support that facilitated practitioners' skill development	(Francis-Coad et al., 2018)
Context: Opportunities for professional development impacted on<br>*Resource & Reaction:* how staff engaged with EBP<br>*Outcome:* that informed the quality of the staff and patient experience	(Stalpers *et al.*, 2017)
Context: Poor communication amongst colleagues<br>*Reaction*: hampered HCP uptake of EBP in their everyday practice<br>*Outcome:* that informed the quality of the staff and patient experience	(Stalpers et al., 2017)
Context: Fostering a social environment that prioritises an intervention as a standing item of business<br>*Reaction*: improves attention to practice<br>*Outcome*: and fosters action in practice	(Stalpers et al., 2017)

Context: Organisations that invest in environmental resources<br>*Resource:* foster better readiness to implement change<br>*Outcome:* leading to more successful implementation	(Stalpers et al., 2017)
Context: Organisations that invest in system supports and resources<br>*Reaction:* better support clinicians<br>*Outcome:* in the implementation of an intervention	(Stalpers et al., 2017)
Context: Excessive staff workload<br>*Reaction:* fosters lack of attention on intervention<br>*Outcome*: and leads to loss of intervention fidelity	(Stalpers et al., 2017)
Context: Competing initiatives<br>*Reaction:* fosters feelings of confusion<br>*Outcome:* leading to an organisational barrier to implementation	(Stalpers et al., 2017)
Context: Inconsistent information and knowledge gaps regarding mental health care model planning and implementation<br>*Reaction:* does not facilitate full engagement in the care-planning process<br>*Outcome:* results in less adoption and fidelity to the model	(Sims *et al.*, 2018)
Context: Negative impact of organisation's efforts to meet implementation strategy targets is<br>*Reaction:* staff feeling watched , under scrutiny, being tested, having extra paper work and being under time pressure<br>*Outcome:* results in disengagement	(Sims et al., 2018)
Context: Fostering stakeholder engagement through<br>*Resource:* timely effective 'low tech' feedback, recognition of existing expertise, motivation, communication and education<br>*Outcome:* facilitates ownership and participation leading to participation in initiative	(Sims et al., 2018)
Context: Intermediaries that provide high level physical presence on clinical units<br>*Resource:* act as a reminder for frontline staff of their practice<br>*Outcome:* and promote prompt change to adhere evidence based practice.	(Sims et al., 2018)
Context: Intermediaries that conduct practice-based teaching<br>*Resource:* meet front line staff learning needs in a meaningful way<br>*Outcome:* and results in more knowledgeable and skilled staff.	(Sims et al., 2018)
Context: Intermediaries that use facilitative approaches giving feedback (discretely and in context) for performance processes<br>*Reaction:* prompt front line staff to consider their practice<br>*Outcome:* and modify in accordance with evidence	(Sims et al., 2018)
Context: Complex interventions require systematic guidelines accompanied with decision support<br>*Resource:* to enable clinicians gain knowledge and skills in intervention delivery<br>*Outcome:* to commit to change and promote new routine care	(Sims et al., 2018)

Context: Implementation leads and local facilitators are more successful<br>*Resource:* when local staff perceive their feedback is meaningful and making a difference in the implementation planning<br>*Outcome:* leading to change motivation and commitment among staff.	(Sims et al., 2018)
Context: Insufficient existing structures require resourcing for service reorganisation and investment<br>*Resource:* to ensure organisational & staff capacity and capability<br>*Outcome:* to implement an intervention.	(Sims et al., 2018)
Context: Staff lack of accessibility to pathways and protocols<br>*Resource:* was improved by access to policies and protocols in the settings<br>*Outcome:* Increased uptake and improve stroke management	(Sims et al., 2018)
Context: Working with a busy environment and stressful working conditions<br>*Resource:* MDT cooperation and support and sharing experience<br>*Outcome:* Increased uptake -Communication between departments was believed to play a pivotal role in the success of intervention	(Sims et al., 2018)
Context: Organisational workload environment<br>*Resource:* time for training allowed, change in documentation<br>*Outcome:* Improved knowledge and confidence	(Cross and Cheyne, 2018)
Context: Organisational structure<br>*Resource:* Managerial permission to move to patient-centrered care, reflective approach<br>*Outcome:* less distress for residents, staff satisfaction, family care-giver satisfaction with care	(Cross and Cheyne, 2018)
Context: Unclear responsibilities/boundaries creates tension and discomfort<br>*Resource:* Establishing good working relationships and trust, working as a team<br>*Outcome:* Facilitates better teamwork and implementation	(Cross and Cheyne, 2018)
Context: Intervention program fits with professional mandate/hot topic in policy (credibility of the programme in quality and strength of evidence)<br>*Reaction:* Staff value and see it as a good fit with clinical role<br>*Outcome:* Facilitates program engagement/implementation	(Cross and Cheyne, 2018)
Context: Credible and competent staff working with Risk-Stroke at the stroke unit<br>*Resource:* Time to meet, discuss<br>*Outcome:* Embrace and engage with NQR data; If questioned or unsupported, experienced a decrease of engagement with NQR; if not in stroke unit - less implementation as perceived more work involved	(Cross and Cheyne, 2018)
Context: Relational working builds over time<br>*Reaction:* Shared learning and persistence, time and space allocated, mutual professional development; sense of common endeavour and generated willingness to work together<br>*Outcome:* Becomes embedded and recognised as normal practice; practitioners confident can provide or access services	(Cross and Cheyne, 2018)

Context: Staff resources may be limited: ability to lead, time availability, insufficient knowledge<br>*Resource:* Educate nurses re their new role. Optimize nurse's role by facilitating MDT comprehensive holistic assessment. Create more specialized care transition nursing roles and education to MSc etc<br>*Outcome:* Will empower staff	(Cross and Cheyne, 2018)

**CMOc Feeding into Programme Theory 4**	**Source**
Context: Knowledge deficit on the full potential of falls prevention programme<br>*Resource:* was addressed by available education and training, feedback loop, knowledge sharing and social learning opportunities<br>*Outcome:* enabling the implementation of evidenced based falls prevention strategies	(Cheyne et al., 2013)
Context: Community of Practice member characteristics<br>*Resource*: provided a high level of local knowledge in falls prevention<br>*Outcome*: enabling the implementation of evidenced based falls prevention strategies	(Cheyne et al., 2013)
Context: Knowledge deficit on the full potential of NSO data was addressed by<br>*Resource:* education and training on NSOs for staff<br>*Outcome:* resulting in nurses' understanding the implications of data for benchmarking and use nationally to inform patient outcomes	(Cheyne et al., 2013)
Context: Focus on an outcome of reducing unplanned admissions<br>*Reaction*: clashed with staffs' professional values (supporting person-centred care) and patient-focused outcomes<br>*Outcome*: leading to uncertainty over boundaries of care and creating tension and burden for staff.	(Francis-Coad et al., 2018)
Context: Congruence at both strategic and practitioner levels<br>*Resource:* that took account of practitioners values and goals<br>*Outcome:* led to efficient implementation were present, and piecemeal implementation were absent	(Francis-Coad et al., 2018)
Context: Practitioners had an understanding of, and skills in mental health care<br>*Resource* & *Reaction*: facilitated by their reflection on their own practice<br>*Outcome*: leading to the examination of assumptions that underpin practise that could inform practitioners' relationship-building	(Francis-Coad et al., 2018)
Context: Staff were knowledgeable in use of asthma guidelines<br>*Reaction:* but some staff used them and some did not<br>*Outcome:* which informed the optimization of the PCAPP prior to its evaluation	(Francis-Coad et al., 2018)
Context: Social and professional identity<br>*Resource*: facilitated use of a multidisciplinary approach<br>*Outcome*: which informed the optimization of the PCAPP prior to its evaluation	(Francis-Coad et al., 2018)
Context: Social influences	(Francis-Coad et al.,

Reaction: affected staffs approach to PCAPP<br>*Outcome:* which informed the optimization of the PCAPP prior to its evaluation	2018)
Context: Social influences<br>*Reaction*: affected staff concerns over patient use of PCAPP<br>*Outcome*: which informed the optimization of the PCAPP prior to its evaluation	(Francis-Coad et al., 2018)
Context: Champions that are trusting and have credibility<br>*Reaction:* generate a tension for change<br>*Outcome:* leading to better implementation commitment	(Sims et al., 2018)
Context: Organisation with a presence of an intermediary<br>*Resource*: foster an environment of rapport and trust<br>*Outcome*: leading to a collegiate atmosphere, better teamwork and improvements in QI	(Sims et al., 2018)
Context: Staff lack of education and awareness of evidence-based treatment (stroke)<br>*Resource & Reaction:* were provided with training and education and also included CPD<br>*Outcome:* that resulted in improved stroke care by increased uptake of evidence-based treatment	(Sims et al., 2018)
Context: Beliefs about consequences of intervention<br>*Reaction*: stemmed from old- fashioned views on stroke, belief re positive outcomes not present<br>*Outcome*: Reluctance to administer intervention	(Sims et al., 2018)
Context: Midwives need to be in control of their own workload to be able to practise flexibly and autonomously<br>*Resource:* Prioritising space & time for team meetings-a shared vision and commitment across the workforce<br>*Outcome:* Role satisfaction	(Sims et al., 2018)
Context: Unclear responsibilities/boundaries creates tension and discomfort<br>*Resource*: Establishing good working relationships and trust, working as a team<br>*Outcome*: Facilitates better teamwork and implementation	(Cross and Cheyne, 2018)
Context: Local stakeholders knowledge of and interest in stroke and QI<br>*Resource:* Facilitated learning about the registry and stroke care. Can apply the NQR data to effectively initiate, carry out, and evaluate quality improvement<br>*Outcome:* Promotes implementation of local quality improvement initiatives	(Cross and Cheyne, 2018)
Context: Beliefs of staff in relation to end of life care: curative versus palliative care<br>*Resource*: Education, training and support<br>*Outcome*: Motivated staff to improve care, sustained implementation; increased competence and capacity; new competencies and beliefs among staff on why and how to use the pathway. Education created more confidence and openness in staff to discuss death and dying. This in turn improved communication and collaboration amongst the MDT and with patients and their relatives.	(Cross and Cheyne, 2018)

**CMOc Feeding into Programme Theory 5**	**Source**
Context: Support for programme at national and local level<br><br>*Resource*: facilitated by the presence of resources and local champions<br>*Outcome*: enabled midwifes to focus on implementation	(Cheyne et al., 2013)
Context: Senior management investment in giving staff time<br>*Reaction*: motivated staff to participate<br>*Outcome:* enabling the implementation of evidenced based falls prevention strategies	(Francis-Coad et al., 2018)
Context: Staff receptivity to falls prevention programme<br>*Resource*: was facilitated by available education and training, feedback loop, knowledge sharing and social learning opportunities<br>*Outcome*: enabling the implementation of evidenced based falls prevention strategies	(Francis-Coad et al., 2018)
Context: Knowledge deficit on the full potential of falls prevention programme<br>*Resource* & *Reaction*: was addressed by available education and training, feedback loop, knowledge sharing and social learning opportunities<br>*Outcome:* enabling the implementation of evidenced based falls prevention strategies	(Francis-Coad et al., 2018)
Context: Clarity of local and national policies was facilitated by<br>*Resource*: education and training on NSOs for staff<br>*Outcome*: resulting in nurses' understanding the implications of data for benchmarking and use nationally to inform patient outcomes	(Stalpers et al., 2017)
Context: Cohesive message on standards and practice between study sites was facilitated by<br>*Resource*: education and training on NSOs for staff<br>*Outcome*: so that NSOs were not seen as additional work and burden	(Stalpers et al., 2017)
Context: Knowledge deficit on the full potential of NSO data was addressed by<br>*Resource*: education and training on NSOs for staff<br>*Outcome:* resulting in nurses' understanding the implications of data for benchmarking and use nationally to inform patient outcomes	(Stalpers et al., 2017)
Context: Availability of staff education and training<br>*Resource*: facilitated awareness of physical and psychological patient care needs<br>*Outcome*: enabling ward managers to audit the standard of care	(Sims et al., 2018)
Context: Senior management buy in and support for programme facilitated<br>*Resource* & *Reaction*: support from stakeholders<br>*Outcome:* resulting in adherence to principles	(Sims et al., 2018)
Context: Champion and Buddy system in the organisation<br>*Resource* & *Reaction*: meant nurses engaged with the buddies	(Sims et al., 2018)

Outcome: Increasing their engagement with the intentional rounding	
Context: The use of a women's hand held record across maternity services<br>*Resource & Reaction*: emphasised a framework of common assessment, that with training and follow through<br>*Outcome*: might be seen to address the variance in skills	(Cross and Cheyne, 2018)
Context: Different stages of training and implementation across health boards<br>*Reaction*: led to correspondingly different approaches to patient assessment by Midwifes<br>*Outcome:* that resulted in the midwives experiential learning being a key factor in motivating longer-term implementation	(Cross and Cheyne, 2018)
Context: An integrated policy<br>*Resource & Reaction*: enabled staff to use standardised guidance documents<br>*Outcome*: leading to Midwifes' adoption of the accessible practice model	(Cross and Cheyne, 2018)
Context: Coordination of the implementation of policy<br>*Resource*: was supported by funded learning materials and change champions<br>*Outcome:* that when present led to advanced training and implementation, and when not present lead to a gap in the capacity to develop strength-based skills	(Cross and Cheyne, 2018)
Context: Facilitators/Champions facilitated and supported clinical practice<br>*Resource* & *Reaction*: which facilitated staff engagement and adherence with infection control standards in practice<br>*Outcome*: leading to adherence with best practice in infection prevention	(Williams, Rycroft-Malone and Burton, 2016)
Context: Facilitators/Champions facilitated and supported clinical practice<br>*Resource*: enabling clinical staff to feel supported in their practice<br>*Outcome*: resulting in increased compliance with Infection control protocols, feelings of collegiality and motivated staff	(Williams et al., 2016)
Context: Facilitators/champions provided practice based education for clinical staff to counteract lack of priority or time for formal training<br>*Reaction*: meaning staff were consistently reminded of the sense of relevance to their own practice<br>*Outcome:* leading to a heightened awareness of infection prevention in clinical areas	(Williams et al., 2016)
Context: Facilitators/Champions were present<br>*Resource*: who staff engaged with<br>*Outcome*: resulting in staff adopting and embedding new practices	(Avra *et al*., 2018)
Context: Passive leadership endorsement<br>*Reaction*: discouraged staff engagement<br>*Outcome:* leading to variability in adoption of new practices	(Avra et al., 2018)
Context: Low morale and high staff turnover<br>*Reaction*: discouraged staff engagement<br>*Outcome*: leading to variability in adoption of new practices	(Avra et al., 2018)

Context: High workload and competing demands<br>*Reaction*: discouraged staff engagement<br>*Outcome*: resulting in lack of engagement with new practices	(Avra et al., 2018)
Context: Where the QI strategy was supported by the National Implementation Research Network<br>*Resource* & *Reaction*: staff engaged with provided resources (e.g. implementation logs/champions/electronic reminders and prompts)<br>*Outcome:* resulting in increased chance of intervention success	(Avra et al., 2018)
Context: Variability in available resources<br>*Resource*: led to variability in staff engagement with the intervention<br>*Outcome*: leading to corresponding variation in degree of engagement with intervention	(Avra et al., 2018)
Context: Incorporating recovery into an existing change programme<br>*Resource*: facilitated staff engagement, enthusiasm and change 'receptiveness'<br>*Outcome:* resulting in a sustainable change of practice	(Gee *et al.*, 2017)
Context: Job uncertainty and fear<br>*Reaction*: hindered staff engagement, enthusiasm and change 'receptiveness'<br>*Outcome*: resulting in a lack of sustainable change of practice	(Gee et al., 2017)
Context: Change agents or champions<br>*Resource & Reaction*: encouraged other staffs' engagement, enthusiasm and change 'receptiveness'<br>*Outcome*: resulting in a sustainable change of practice	(Gee et al., 2017)
Context: Organisation had Champion role for new EBP innovations<br>*Resource*: meaning staff engaged with Champions who were 'on the floor', had expertise and were familiar to them<br>*Outcome:* increasing the success of new EBP implementation	(Li *et al.*, 2018)
Context: Context of developing professional expertise derived from critical reflection on implementation of evidence-informed practice<br>*Resource* & *Reaction*: but staff lacked the training and/or confidence in critically assessing the value of this knowledge<br>*Outcome*: resulting in staff potentially undervaluing their individual and collective learning from implementation	(Bryce, Fleming and Reeve, 2018)
Context: Implementation leads<br>*Resource*: assist in identification of clear roles and champion the intervention<br>*Outcome:* fosters change efficacy	(Hanson *et al.*, 2017)
Context: Champions that are trusting and have credibility<br>*Resource* & *Reaction*: generate a tension for change<br>*Outcome*: leading to better implementation commitment	(Wutzke, Benton and Verma, 2016)
Context: Promoting change through stakeholder engagement<br>*Resource & Reaction*: by providing support, having champions, creating opportunities for staff involvement and acknowledging	(Wutzke et al., 2016)

resistance<br>*Outcome*: leading to staff ownership, advocates and maintainers of the change initiative	
Context: Effective implementation leads that engage stakeholders and identify implementation barriers and instigate additional interventions<br>*Resource* & *Reaction*: resolve challenges and meet the need of nurses<br>*Outcome:* making implementation more likely.	(Noyes *et al.*, 2014)
Context: Lack of recognition of Social/professional role and limited social identity, i.e., insufficient recognition by peers and decision makers<br>*Resource* & Reaction: Good leadership -champion identified, verbal persuasion about capability and meetings<br>*Outcome*: Increased uptake of evidence-based management (stroke)	(Craig *et al.*, 2016)
Context: Lack of local champion<br>*Resource*: Availability of local champion<br>*Outcome:* Facilitates program implementation	(Sopcak *et al.*, 2016)
Context: Early engagement of administrators and managers / collaborative and teamwork connections (planning and engaging process)<br>*Resource & Reaction*: Increase stakeholder engagement and optimise communication; with ability to better adapt program to need of participants<br>*Outcome*: Improvement in uptake /desire to join project; clarification of role, scope, expectations	(Sopcak et al., 2016)
Context: Resources e.g. lack of facilitator, time<br>*Resource*: **A dedicated facilitator**: provided training for staff in how to communicate recognition that the patient is dying with both patients and relatives; providing feedback on positive outcomes of pathway usage -using literature; provided reassurance to staff over the withdrawal of unnecessary routine practice such as measuring blood pressure.<br>*Outcome*: Success of LCP: motivated staff; increased confidence in staff to implement the pathway. Facilitation appears to work by making staff aware of the goals of the LCP, providing reassurance in terms of their skills and decision-making in early implementation. This leads to a self-perception of competency and capacity, which increases the likelihood that new approaches are embedded, unnecessary practices are stopped, and communication takes place with relatives and patients.	(McConnell *et al.*, 2013)

**CMOc Feeding into Programme Theory 6**	**Source**
Context: Busy work environment/workload was obviated by<br>*Reaction*: staff who engaged with programme as they saw the benefits for patient care and family communication<br>*Outcome:* which led to increased staff vigilance and awareness of psychological and physical patient needs	(Cheyne et al., 2013)

Context: Availability of staff education and training<br>*Resource*: facilitated awareness of physical and psychological patient care needs<br>*Outcome*: enabling ward managers to audit the standard of care	(Cheyne et al., 2013)
Context: Patients aware of standards of care<br>*Resource* & *Reaction*: empowered patients to nurses visibility of care<br>*Outcome:* Patients have expectation of care and quality of interaction; patient empowerment; visibility of nursing work is clear to all involved in the process of care	(Cheyne et al., 2013)
Context: Partnership working (patient and practitioner)<br>*Resource* & *Reaction*: meant practitioners had time to spend discussing what is important to the patient<br>*Outcome*: leading to patients taking ownership of their health outcomes	(Francis-Coad et al., 2018)
Context: Patients required holistic, personalised support<br>*Resource* & *Reaction*: which enabled them to feel equipped with knowledge and skills<br>*Outcome*: so that they were confident in managing their own health and health outcomes	(Francis-Coad et al., 2018)
Context: Patients were uninformed<br>*Reaction*: as the service had not been developed with, or advertised to, local residents prior to its introduction.<br>*Outcome:* leading to resistance to change from patients and, to a lesser extent, their families.	(Francis-Coad et al., 2018)
Context: Parental knowledge and understanding<br>*Resource & Reaction*: were supported by education but the education was affected by logistics and time constraints<br>*Outcome*: leading to parental uncertainty due to lack of clarify regarding long-term effects of medication	(Francis-Coad et al., 2018)
Context: Divergent views on what services user (SU) and staff (S)want from health care delivery<br>*Resource*: such as wanting involvement in own care (SU) and being diagnosis & health outcomes focused (S)<br>*Outcome:* results in lack of stakeholder consensus and thus implementation failure	(Sims et al., 2018)
Context: Lack of meaningful engagement with service user/ carers by health care professionals<br>*Resource*: with little 'relational' interaction with service user<br>*Outcome*: leads to the lack of satisfaction and involvement by service user	(Sims et al., 2018)
Context: Organisation with a presence of an intermediary<br>*Resource* & *Reaction*: gives an enhanced sense of recognition for patients and relatives<br>*Outcome*: leading to public reassurance	(Sims et al., 2018)
Context: Ability to support and motivate patients<br>*Resource*: Places patient in driver seat to take ownership of roles<br>*Outcome:* Patients feel supported and motivated	(Cross and Cheyne, 2018)
Context: Patient resources may be limited<br>*Reaction*: Determined preferred level of involvement, readiness and capabilities of patients, their family members, and/or caregivers.	(Cross and Cheyne, 2018)

Engaged them in co-design of care transition interventions; educated them. Made aware of follow-up<br>*Outcome*: Will empower patients etc and result in quality care transitions experiences and outcomes	
Context: Perceived need to support patients<br>*Resource & Reaction*: Group meetings. Personalised information. Promoted use of reflective diaries<br>*Outcome:* Patient coping skills increased. Little success with diary	(Cross and Cheyne, 2018)
Context: Perceived need to support newly diagnosed patients separately<br>*Resource & Reaction*: Education focused on managing day-to-day problems and "how to" skills. Use of individual, group and family sessions<br>*Outcome*: Increase in self-efficacy and patients felt more supported in daily life. Additional instruction of family members not effective.	(Cross and Cheyne, 2018)

References

Avra, S., Janet, D., Ian, C., Jacques, L. and Yona, L. (2018) 'Original qualitative research Barriers and facilitators to improving health care for adults with intellectual and developmental disabilities: what do staff tell us?', *Health promotion and chronic disease prevention in Canada,* 38(10), pp. 349-357.

Bryce, C., Fleming, J. and Reeve, J. (2018) 'Implementing change in primary care practice: Lessons from a mixed-methods evaluation of a frailty initiative', *BJGP Open,* 2(1), pp. bjgpopen18X101421-bjgpopen18X101421.

Cheyne, H., Abhyankar, P. and McCourt, C. (2013) 'Empowering change: Realist evaluation of a Scottish Government programme to support normal birth', *Midwifery,* 29(10), pp. 1110-1121.

Craig, L. E., McInnes, E., Taylor, N., Grimley, R., Cadilhac, D. A., Considine, J. and Middleton, S. (2016) 'Identifying the barriers and enablers for a triage, treatment, and transfer clinical intervention to manage acute stroke patients in the emergency department: A systematic review using the theoretical domains framework (TDF)', *Implementation science : IS,* 11(1), pp. 157-157.

Cross, B. and Cheyne, H. (2018) 'Strength-based approaches: a realist evaluation of implementation in maternity services in Scotland', *Journal of public health,* 26(4), pp. 425-436.

Francis-Coad, J., Etherton-Beer, C., Bulsara, C., Blackburn, N., Chivers, P. and Hill, A.-M. (2018) 'Evaluating the impact of a falls prevention community of practice in a residential aged care setting: A realist approach', *BMC health services research,* 18(1), pp. 21-21.

Gee, M., Bhanbhro, S., Cook, S. and Killaspy, H. (2017) 'Rapid realist review of the evidence: achieving lasting change when mental health rehabilitation staff undertake recovery-oriented training', *Journal of advanced nursing,* 73(8), pp. 1775-1791.

Hanson, H. M., Warkentin, L., Wilson, R., Sandhu, N., Slaughter, S. E. and Khadaroo, R. G. (2017) 'Facilitators and barriers of change toward an elder-friendly surgical environment: Perspectives of clinician stakeholder groups', *BMC health services research,* 17(1), pp. 596-596.

Li, S.-A., Jeffs, L., Barwick, M. and Stevens, B. (2018) 'Organizational contextual features that influence the implementation of evidence-based practices across healthcare settings: A systematic integrative review', *Systematic reviews,* 7(1), pp. 72-72.

McConnell, T., O'Halloran, P., Porter, S. and Donnelly, M. (2013) 'Systematic Realist Review of Key Factors Affecting the Successful Implementation and Sustainability of the Liverpool Care Pathway for the Dying Patient', *Worldviews on evidence-based nursing,* 10(4), pp. 218-237.

Noyes, J., Lewis, M., Bennett, V., Widdas, D. and Brombley, K. (2014) 'Realistic nurse-led policy implementation, optimization and evaluation: novel methodological exemplar', *Journal of advanced nursing,* 70(1), pp. 220-237.

Sims, S., Leamy, M., Davies, N., Schnitzler, K., Levenson, R., Mayer, F., Grant, R., Brearley, S., Gourlay, S., Ross, F. and Harris, R. (2018) 'Realist synthesis of intentional rounding in hospital wards: exploring the evidence of what works, for whom, in what circumstances and why', *BMJ quality & safety,* 27(9), pp. 743-752.

Sopcak, N., Aguilar, C., O'Brien, M. A., Nykiforuk, C., Aubrey-Bassler, K., Cullen, R., Grunfeld, E. and Manca, D. P. (2016) 'Implementation of the BETTER 2 program: A qualitative study exploring barriers and facilitators of a novel way to improve chronic disease prevention and screening in primary care', *Implementation science : IS,* 11(1), pp. 158-158.

Stalpers, D., De Vos, M. L. G., Van Der Linden, D., Kaljouw, M. J. and Schuurmans, M. J. (2017) 'Barriers and carriers: a multicenter survey of nurses' barriers and facilitators to monitoring of nurse-sensitive outcomes in intensive care units', *Nursing open,* 4(3), pp. 149-156.

Williams, L., Rycroft-Malone, J. and Burton, C. R. (2016) 'Implementing best practice in infection prevention and control. A realist evaluation of the role of intermediaries', *International journal of nursing studies,* 60, pp. 156-167.

Wutzke, S., Benton, M. and Verma, R. (2016) 'Towards the implementation of large scale innovations in complex health care systems: Views of managers and frontline personnel', *BMC research notes,* 9(1), pp. 327-327.
